# Supplementary material for: Insight on a Competitive Nucleophilic Addition Reaction of Nε-(Carboxymethyl) Lysine or Different Amino Acids with 4-Methylbenzoquinone
Source: Foods. 2022 May 13;11(10):1421. doi: 10.3390/foods11101421 (PMC9140783; doi:10.3390/foods11101421)
Supplement: Supplementary file 1 [file foods-11-01421-s001.zip › Supplementary material .pdf]

Supplementary Materials

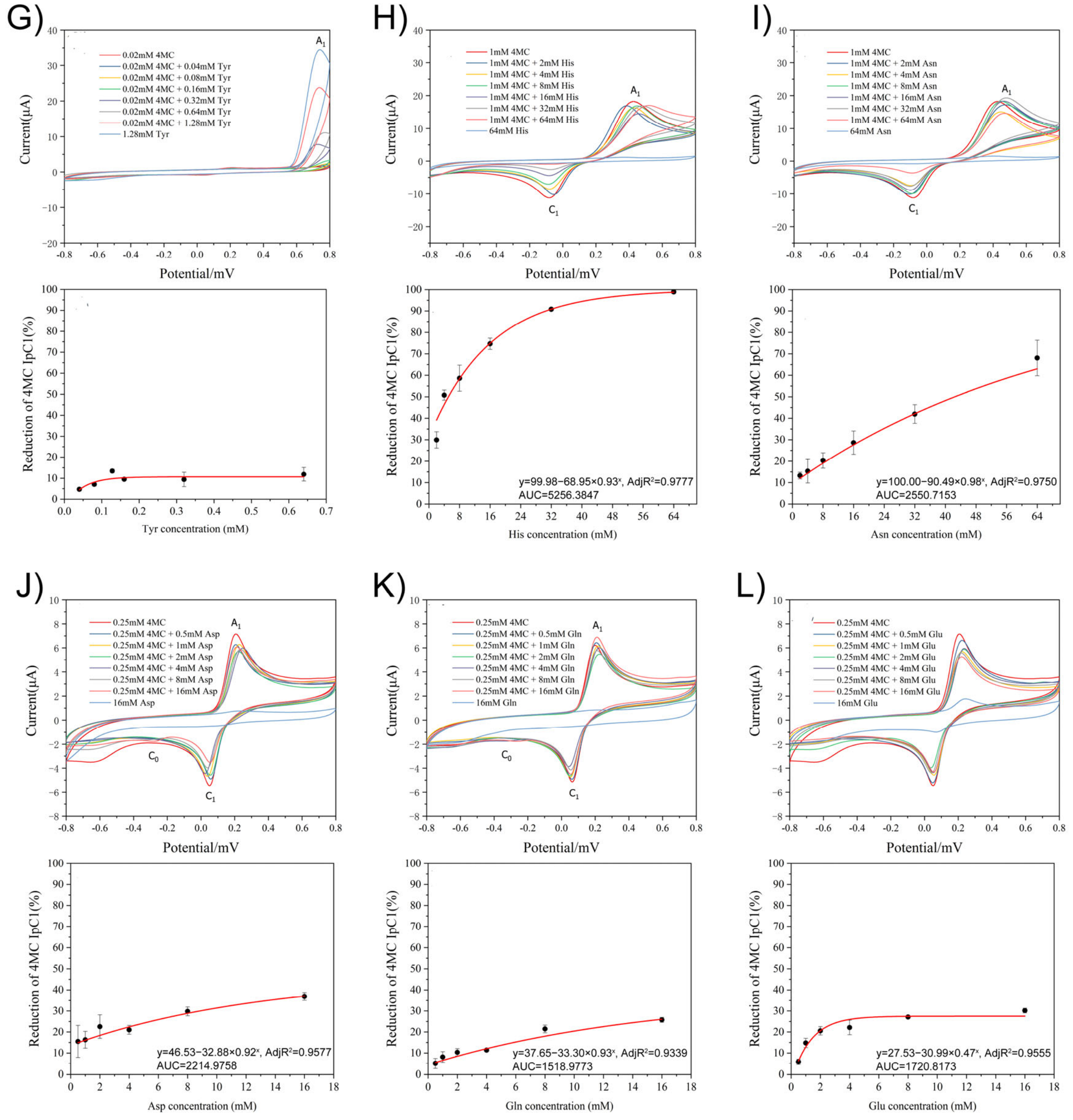

Figure S1. Cyclic voltammograms and fitted curve of 4MC (1mM) in the presence of different concentrations of nucleophiles (G: Tyr; H: his; I: Asn; J: Asp; K: Gln; L: Glu) at the surface of glass carbon electrode in 0.2 M phosphate buffer (pH 7.4). Scan rate of 10 mV/s. Temperature of 25 ± 1 °C.

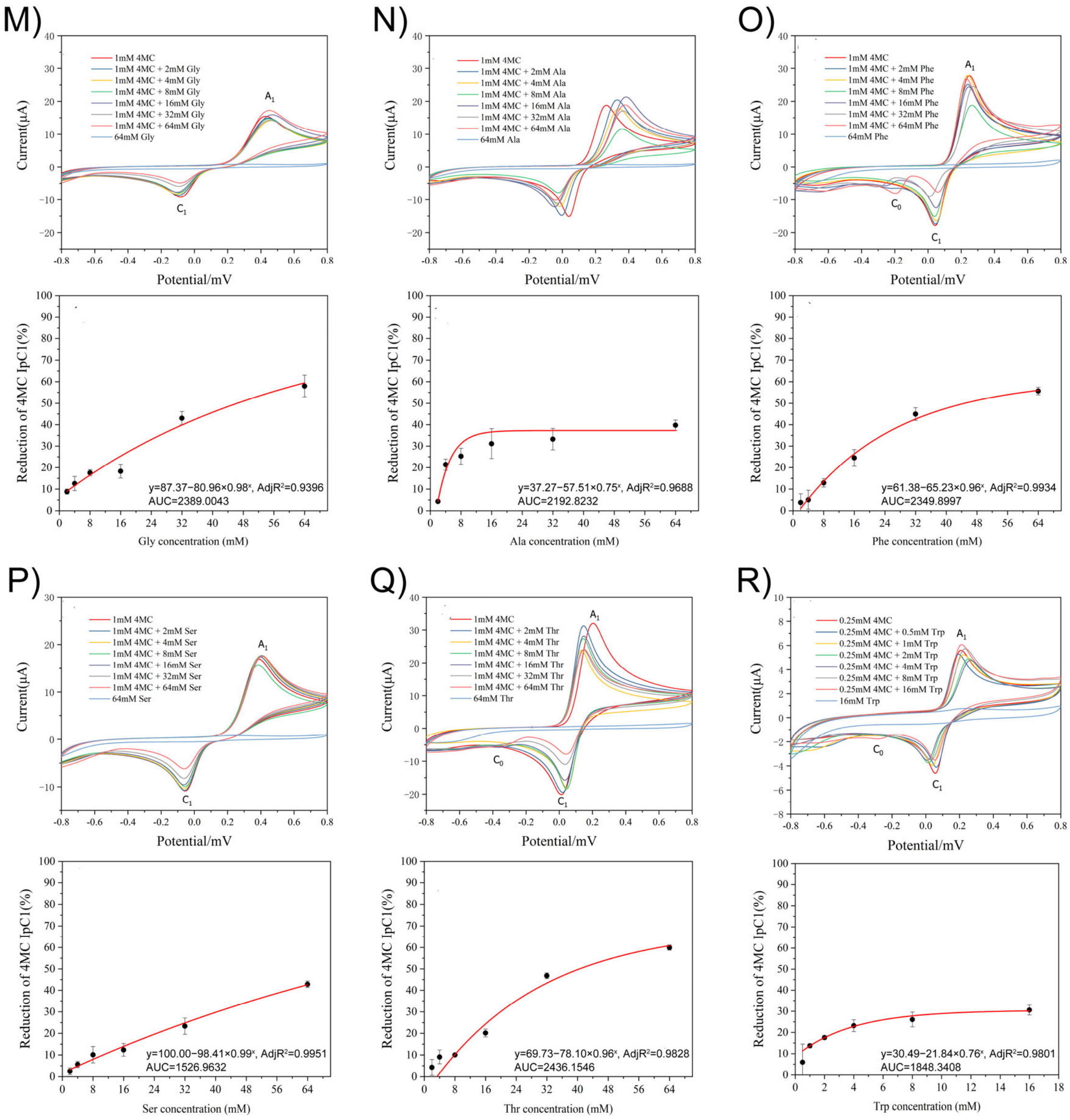

Figure S2. Cyclic voltammograms and fitted curve of 4MC (1mM) in the presence of different concentrations of nucleophiles (M: Gly; N: Ala; O: Phe; P: Ser; Q: Thr; R: Trp) at the surface of glass carbon electrode in 0.2 M phosphate buffer (pH 7.4). Scan rate of 10 mV/s. Temperature of 25 ± 1 °C.

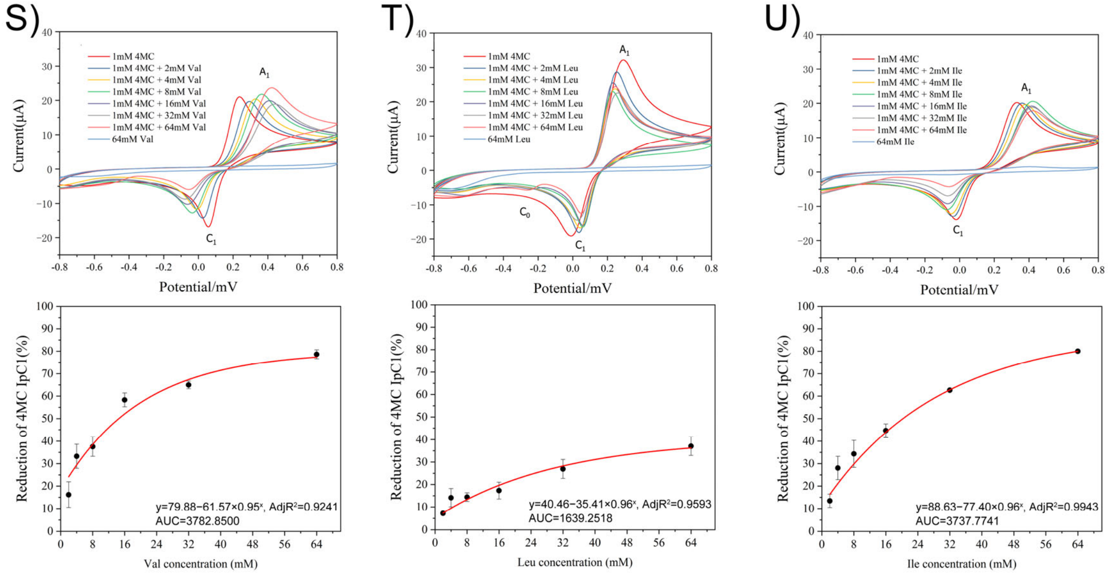

Figure S3. Cyclic voltammograms and fitted curve of 4MC (1mM) in the presence of different concentrations of nucleophiles (S: Val; T: Leu; U: Ile) at the surface of glass carbon electrode in 0.2 M phosphate buffer (pH 7.4). Scan rate of 10 mV/s. Temperature of 25 ± 1 °C.

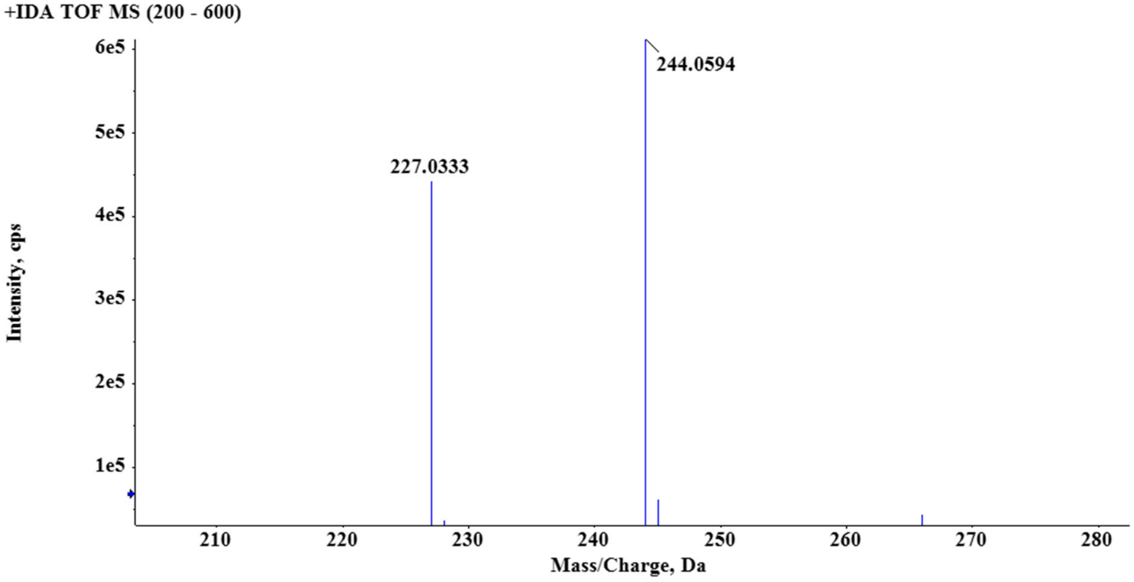

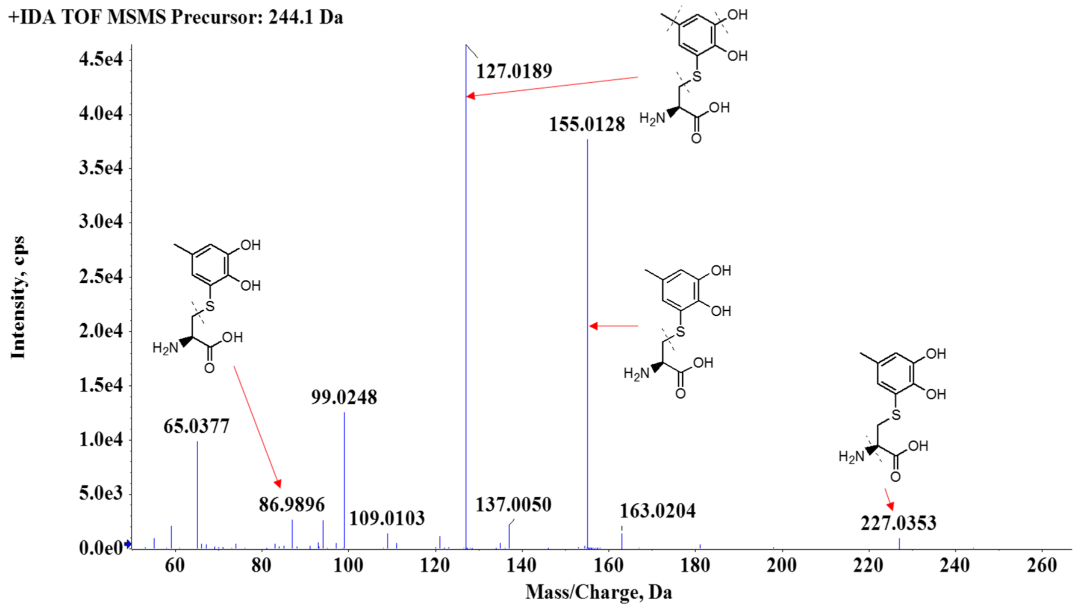

Figure S4. Mass spectra, proposed structures and corresponding fragmentation pathways of L-Cys-4-MC adduct.

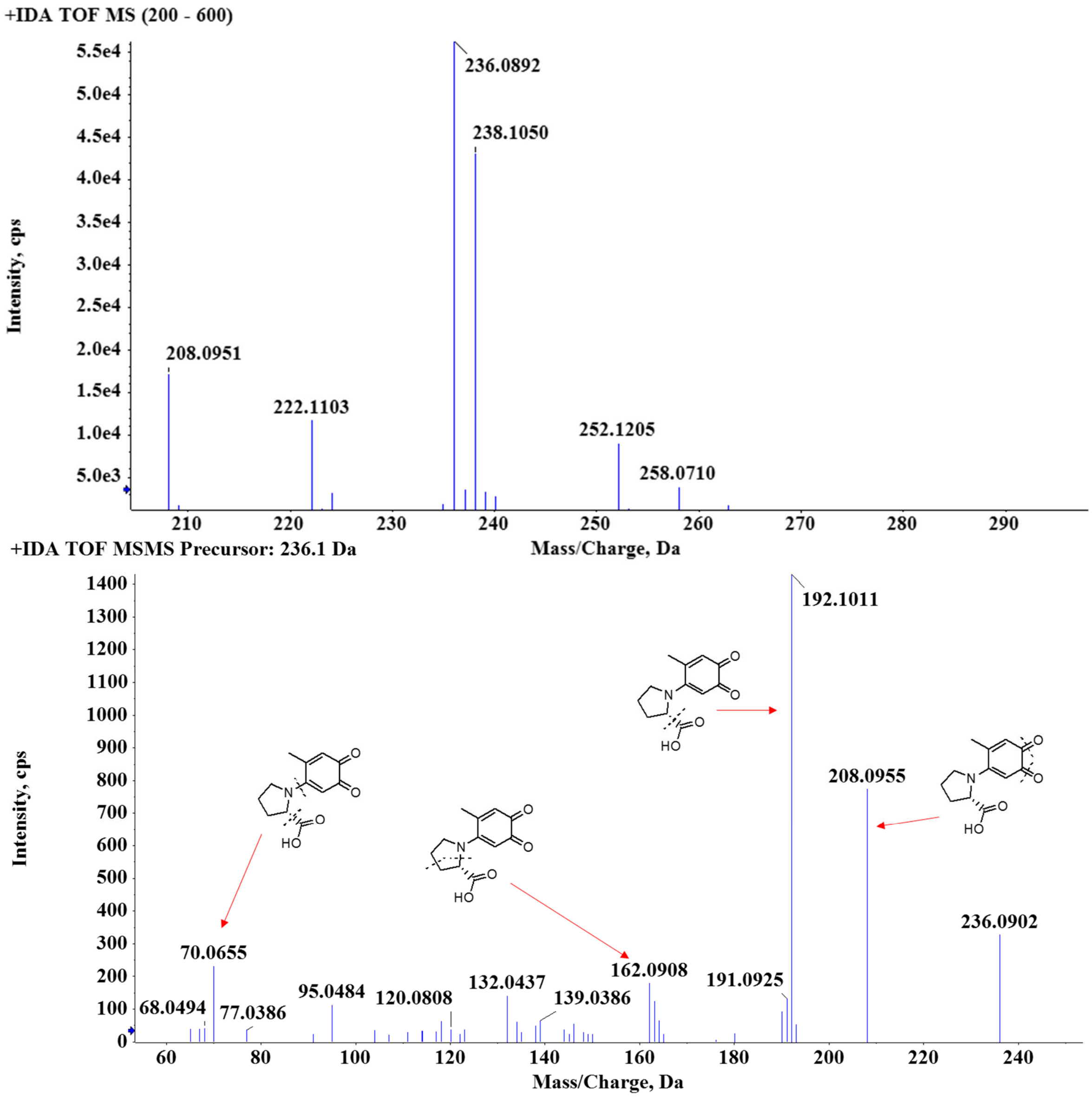

Figure S5. Mass spectra, proposed structures and corresponding fragmentation pathways of L-Pro-4-MBQ adduct.

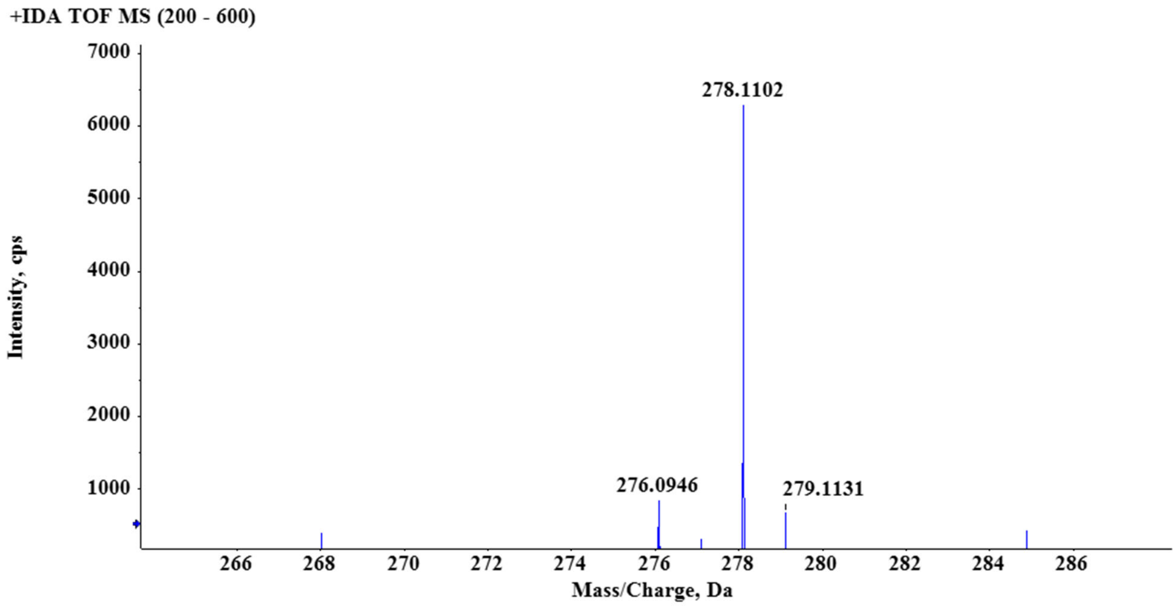

+IDA TOF MS (200 - 600)
Intensity, cps
Mass/Charge, Da
278.1102
276.0946
279.1131

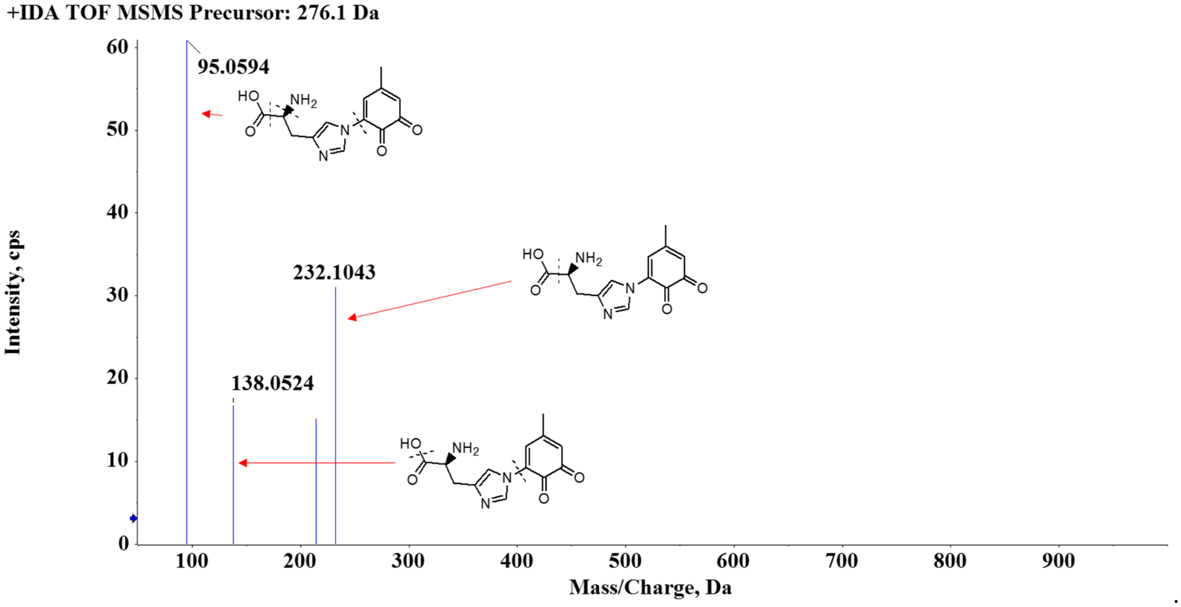

+IDA TOF MSMS Precursor: 276.1 Da
Intensity, cps
Mass/Charge, Da
95.0594
232.1043
138.0524

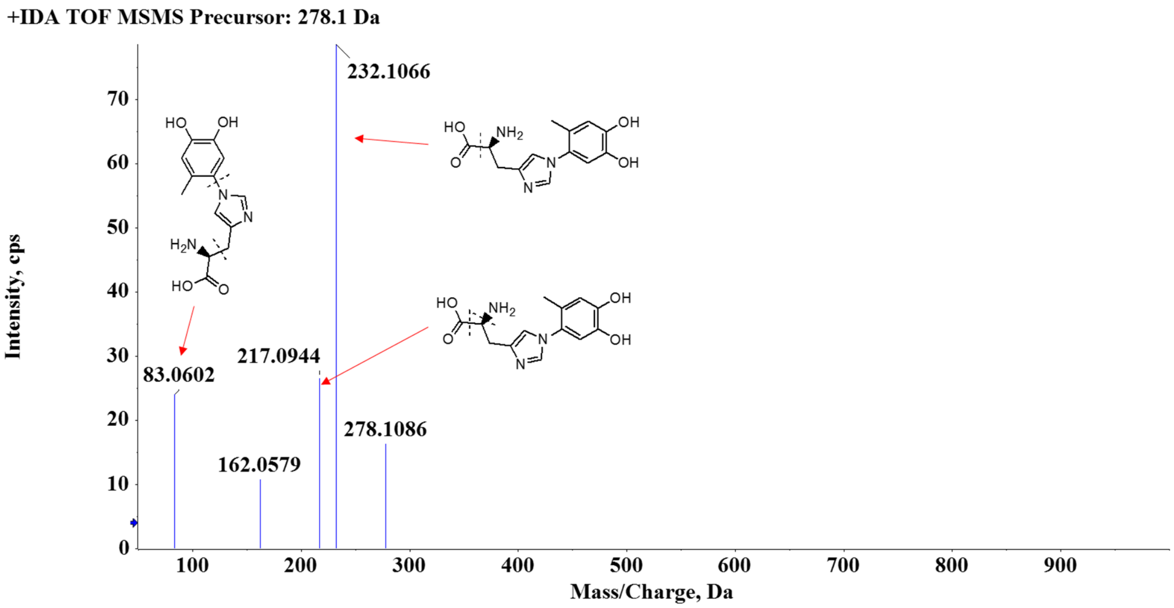

Figure S6. Mass spectra, proposed structures and corresponding fragmentation pathways of L-His-4-MBQ and L-His-4-MC adduct.

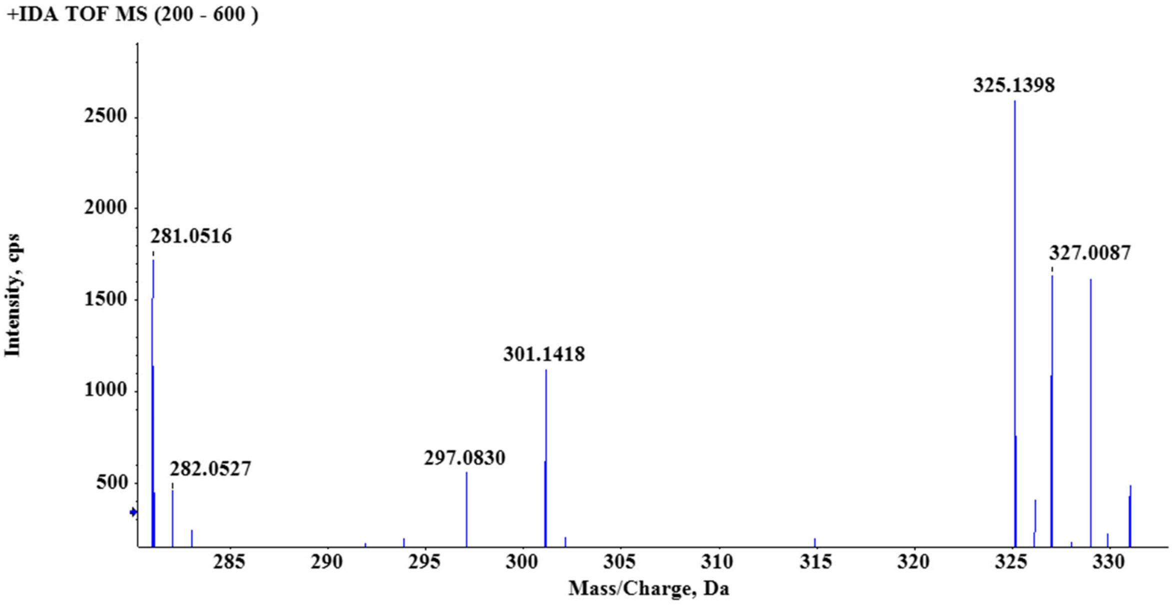

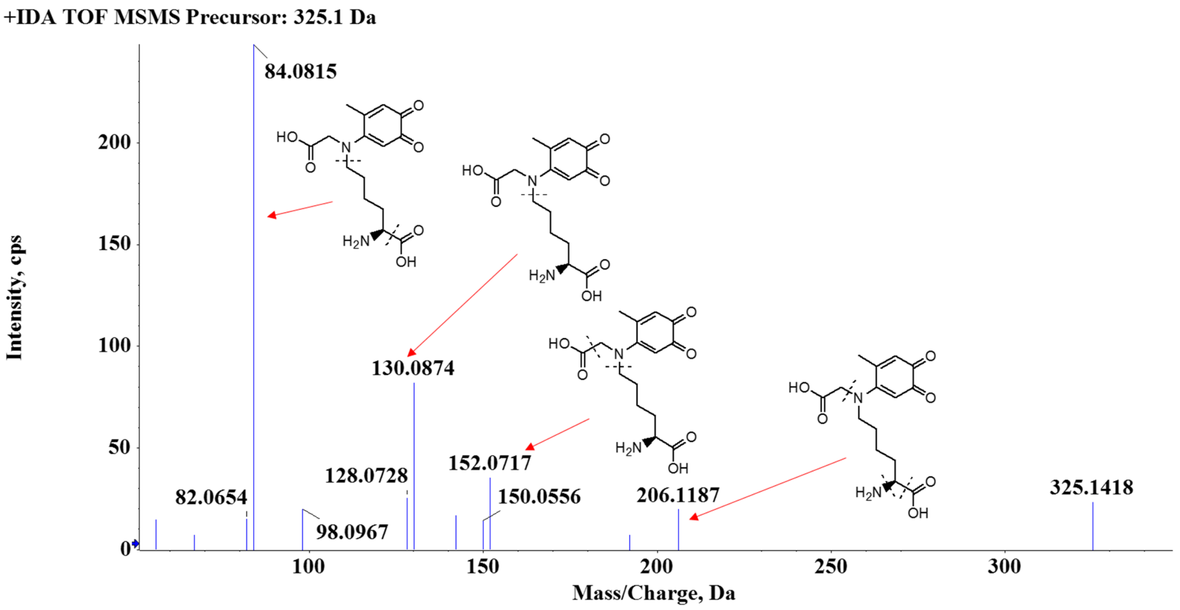

Figure S7. Mass spectra, proposed structures and corresponding fragmentation pathways of CML-4-MBQ adduct.

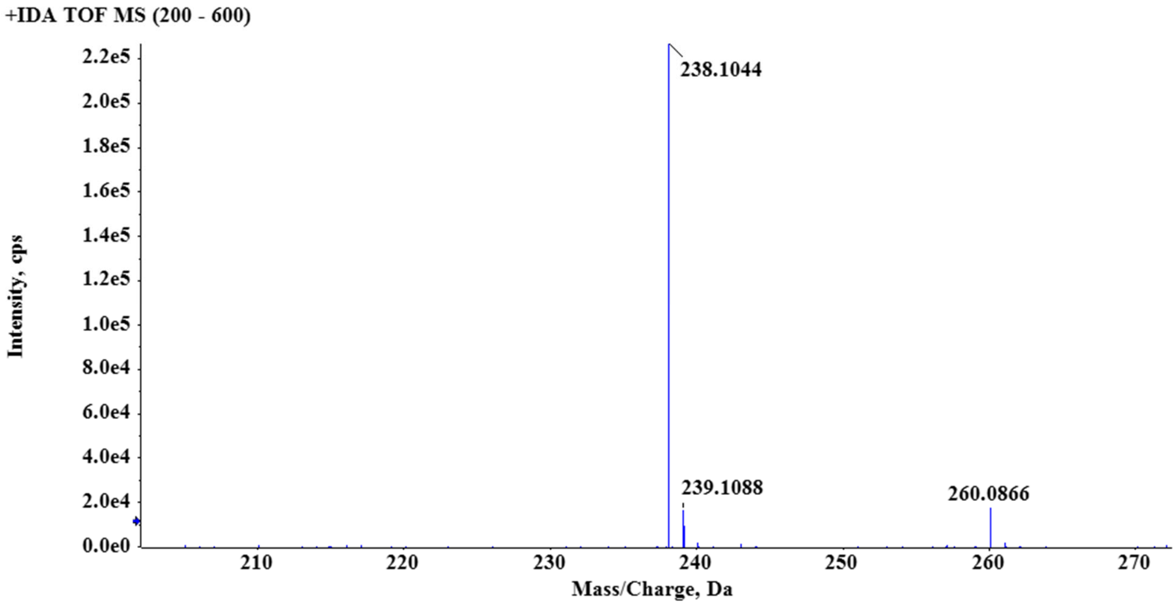

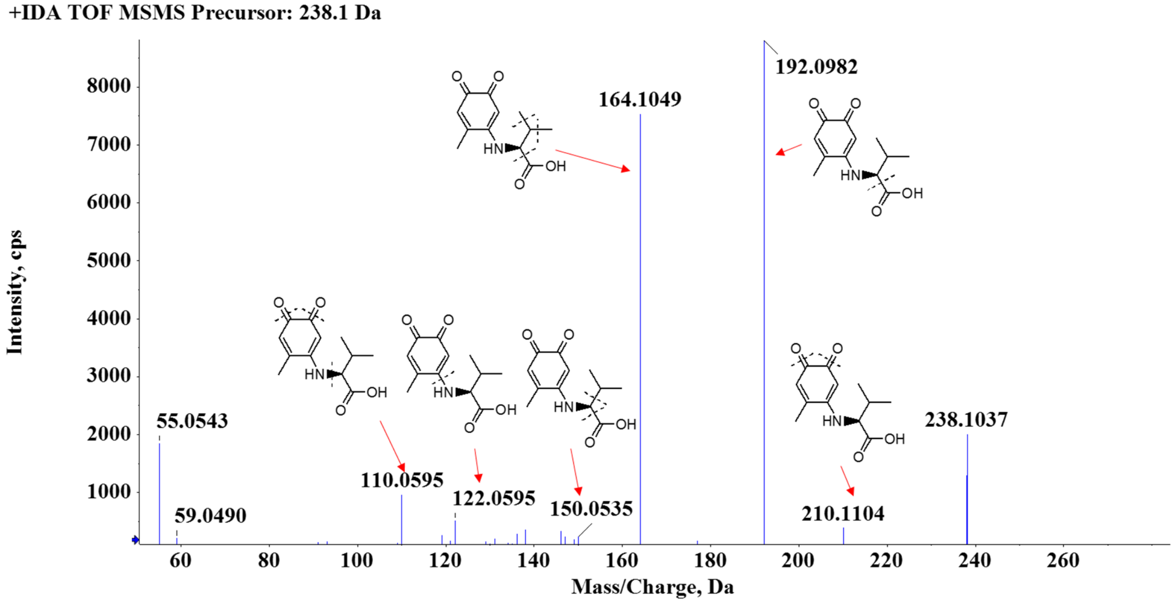

Figure S8. Mass spectra, proposed structures and corresponding fragmentation pathways of L-Val-4-MBQ adduct.

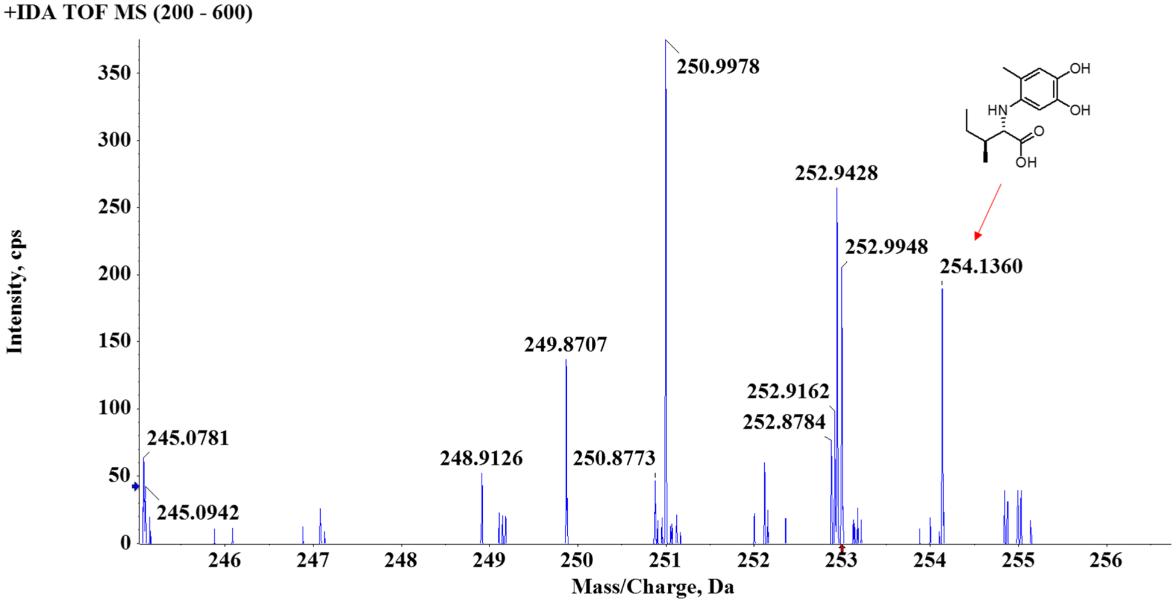

Figure S9. Mass spectra, proposed structures of L-Ile-4-MC adduct.

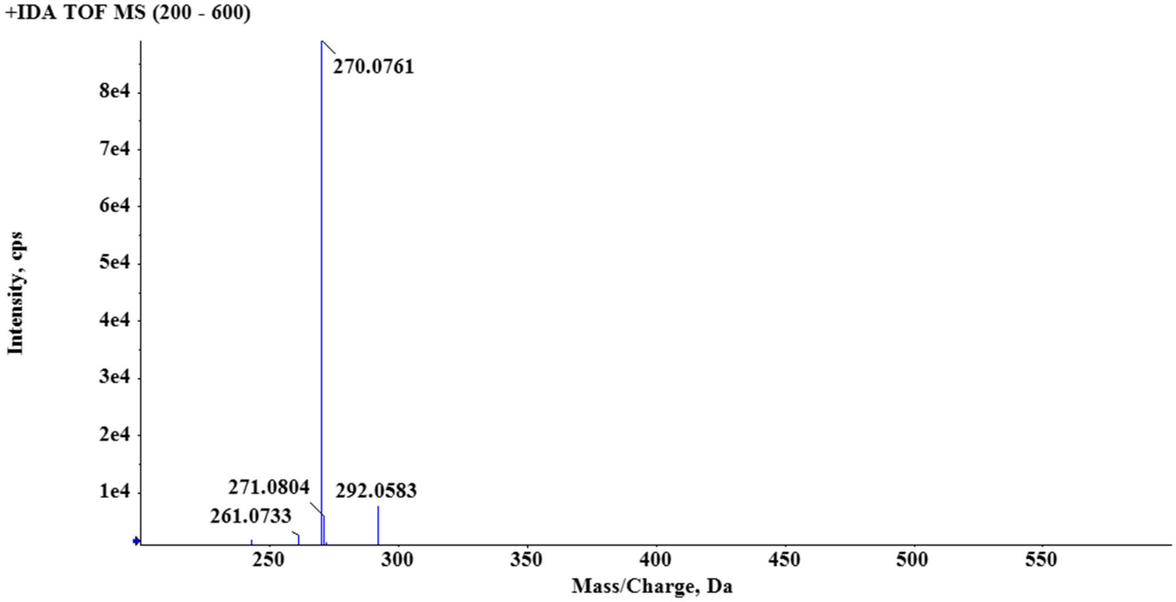

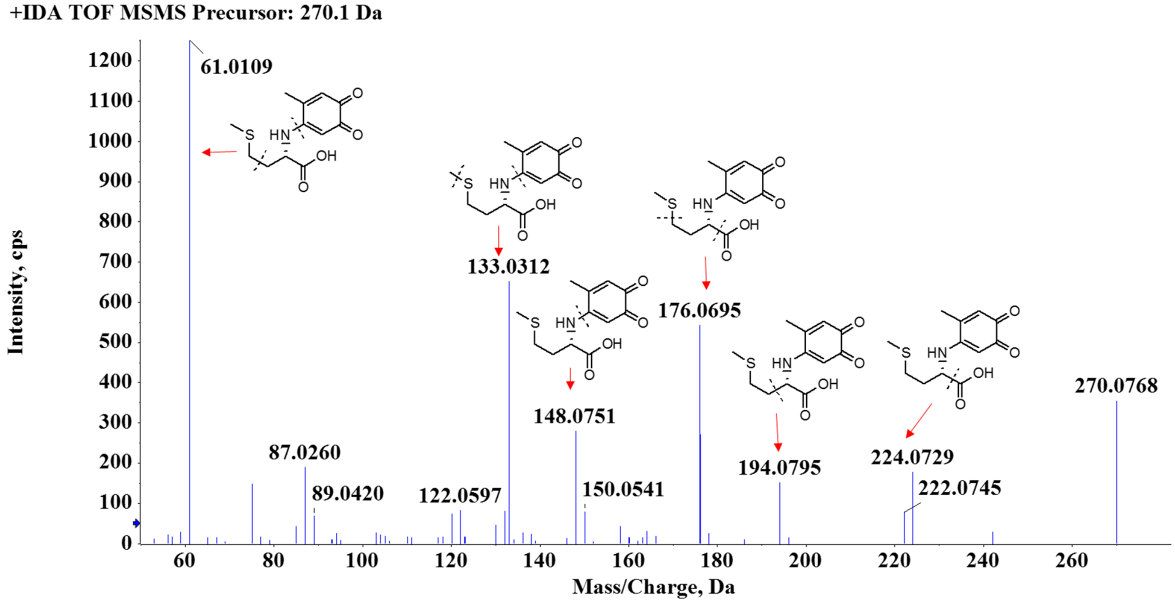

Figure S10. Mass spectra, proposed structures and corresponding fragmentation pathways of L-Met-4-MBQ adduct.

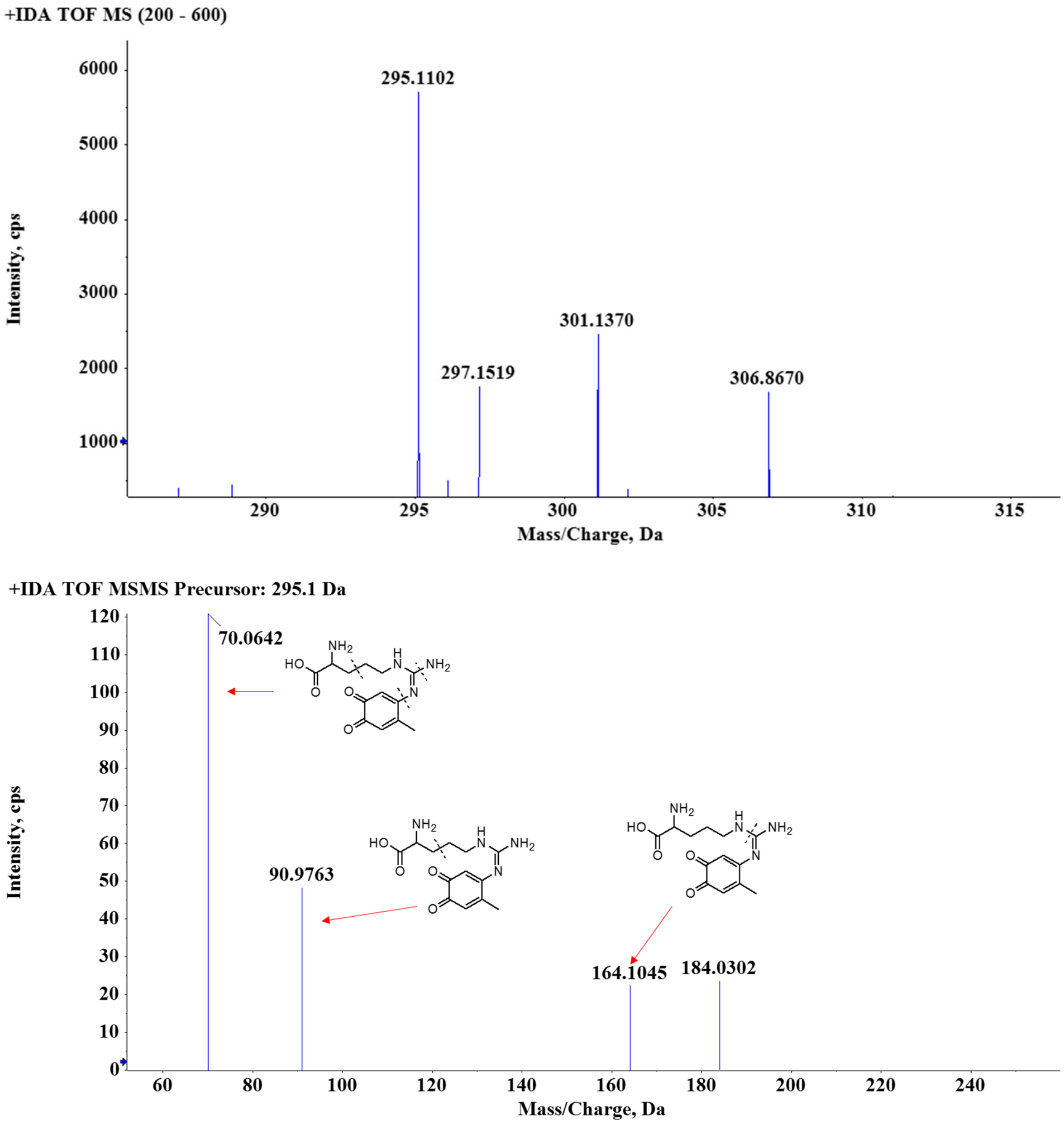

Figure S11. Mass spectra, proposed structures and corresponding fragmentation pathways of L-Arg-4-MBQ adduct.

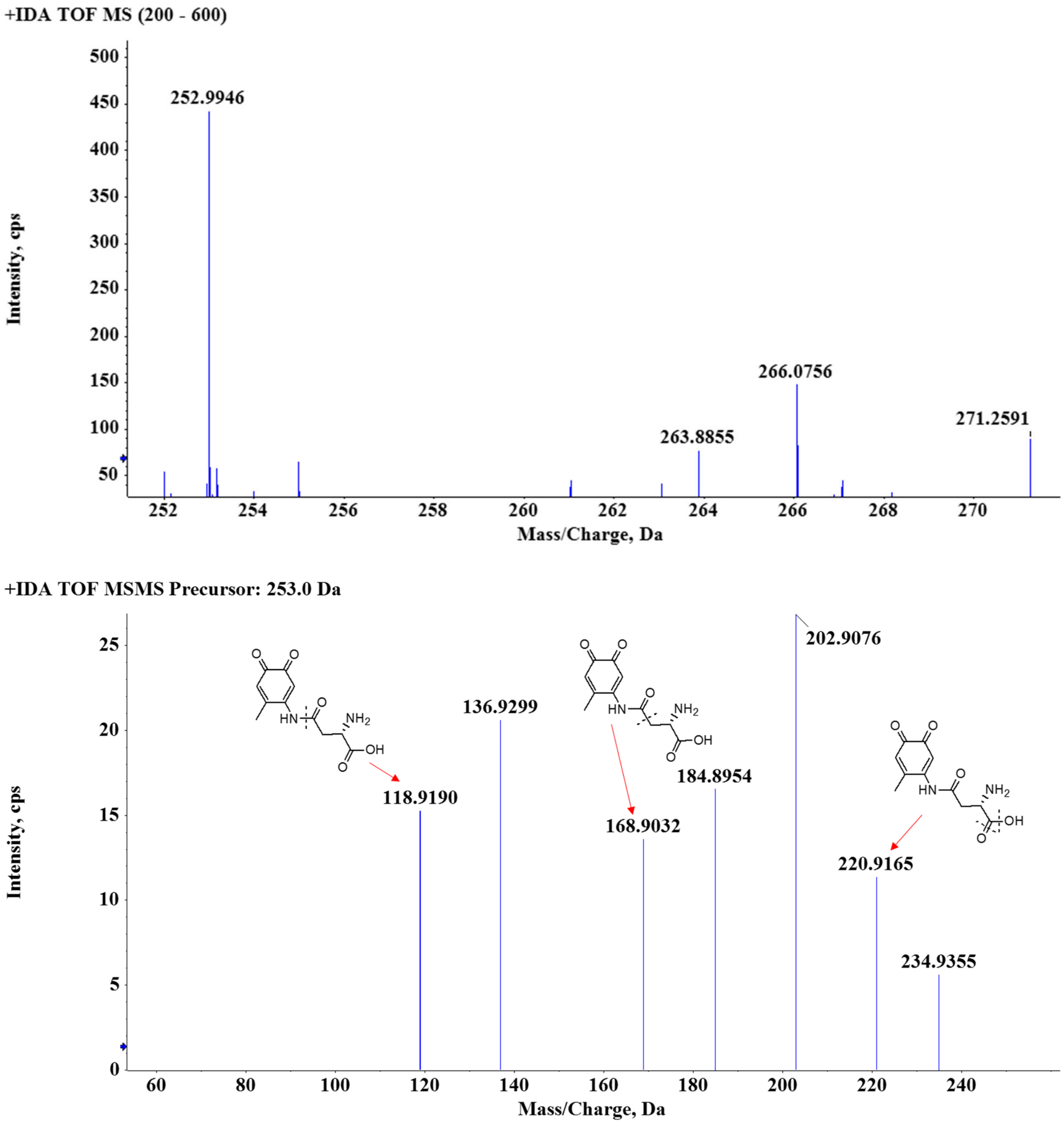

Figure S12. Mass spectra, proposed structures and corresponding fragmentation pathways of L-Asn-4-MBQ adduct.

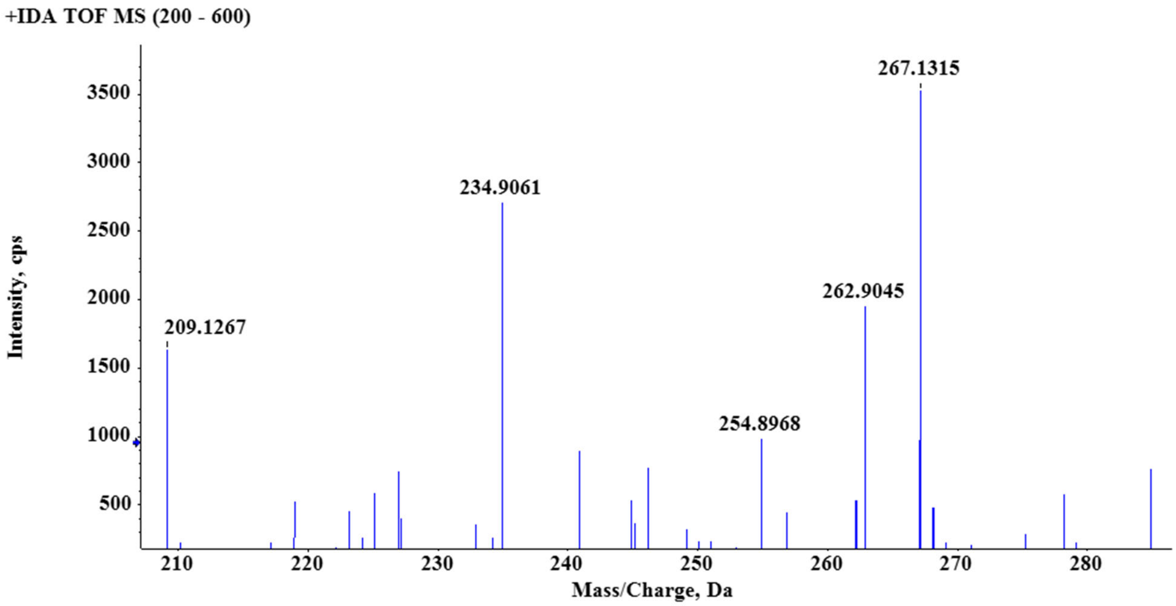

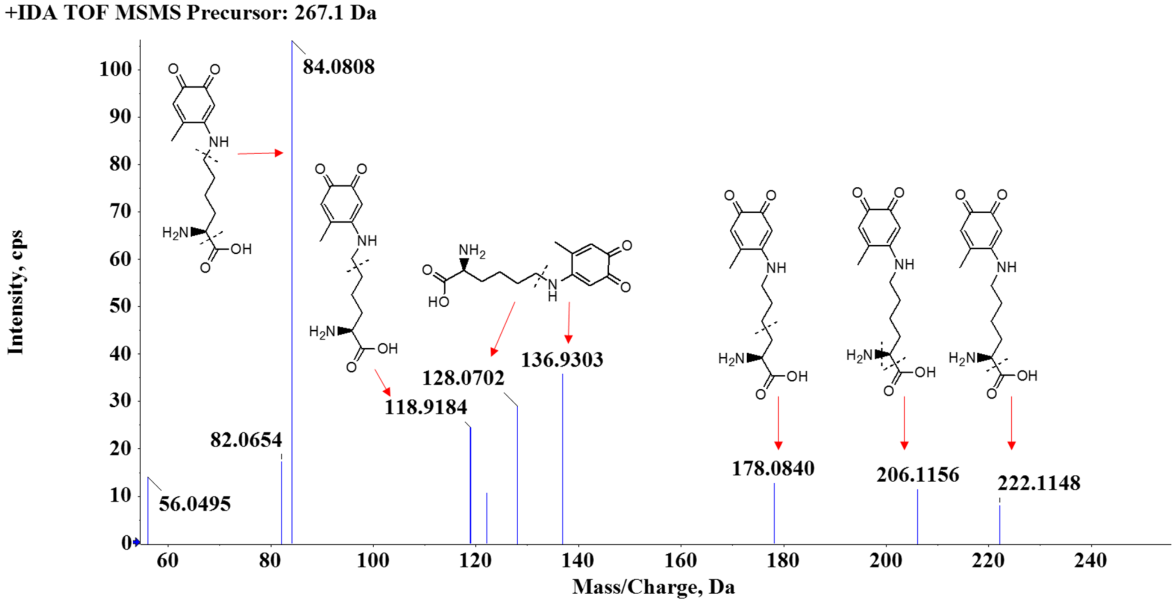

Figure S13. Mass spectra, proposed structures and corresponding fragmentation pathways of L-Lys-4-MBQ adduct.

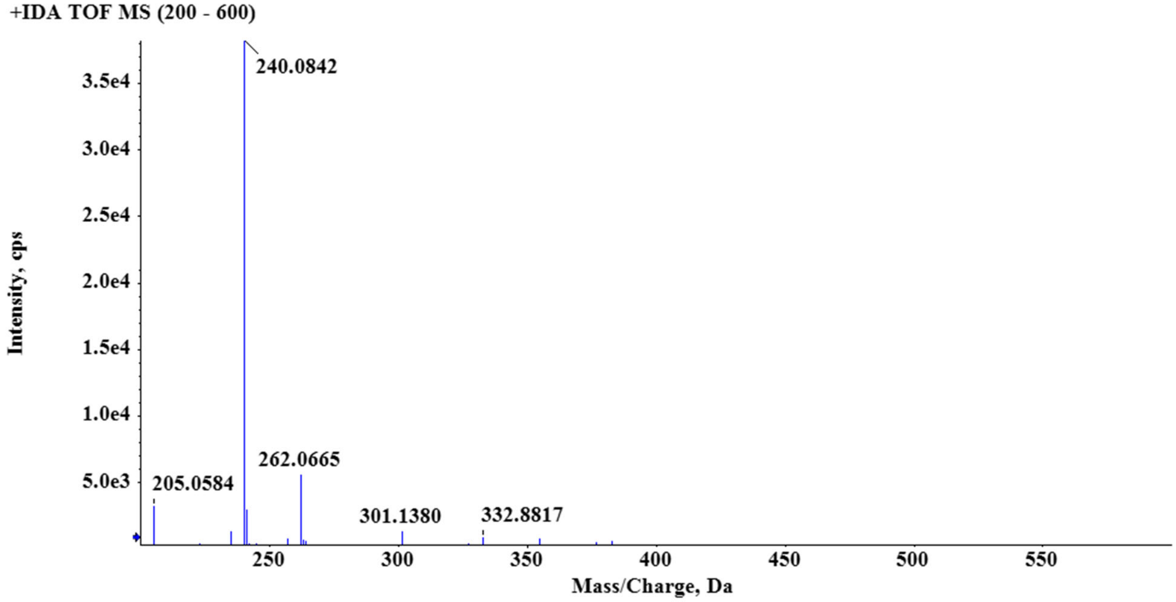

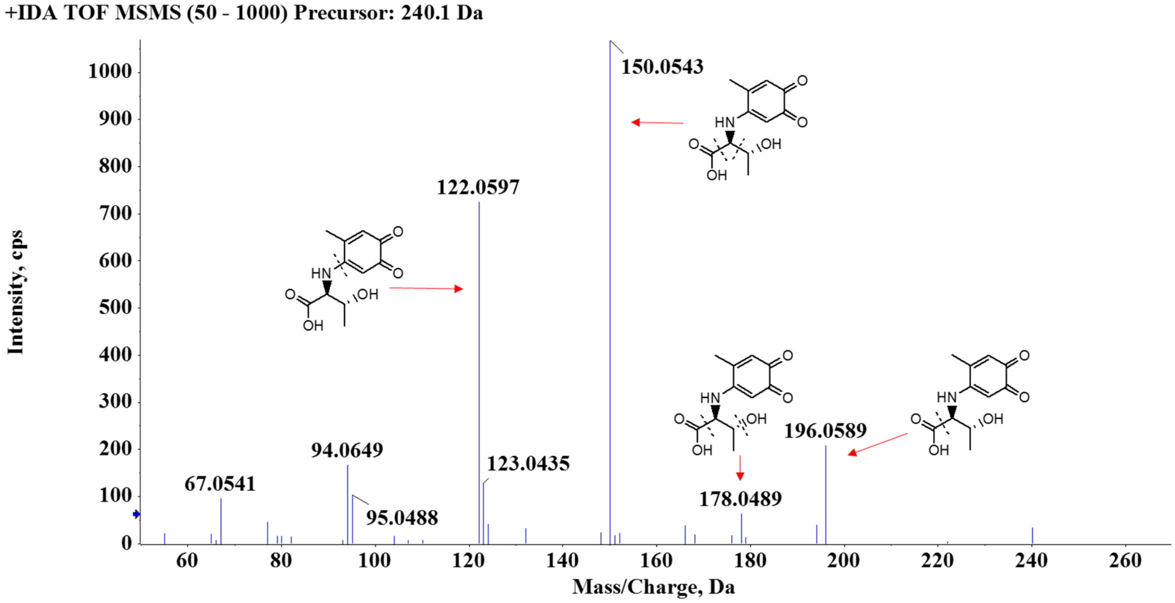

Figure S14. Mass spectra, proposed structures and corresponding fragmentation pathways of L-Thr-4-MBQ adduct.

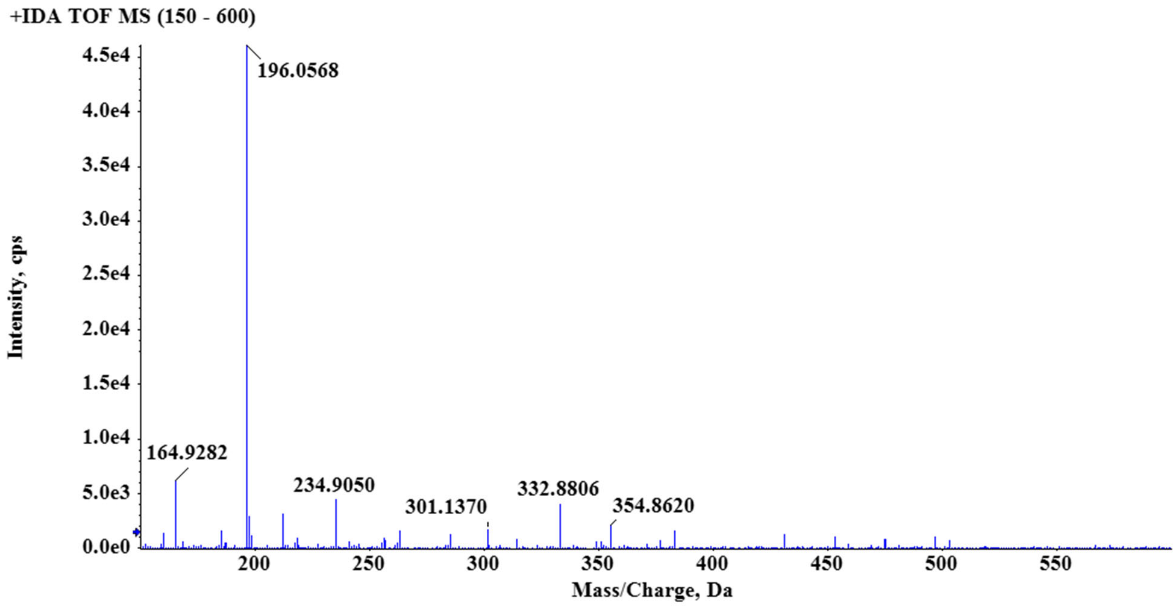

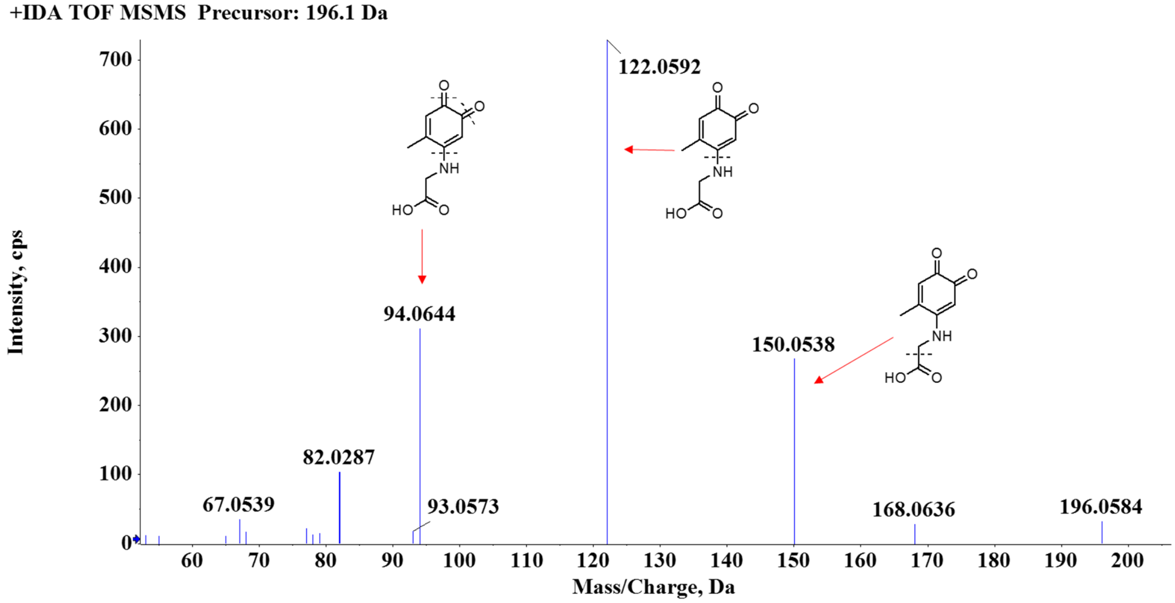

Figure S15. Mass spectra, proposed structures and corresponding fragmentation pathways of Gly-4-MBQ adduct.

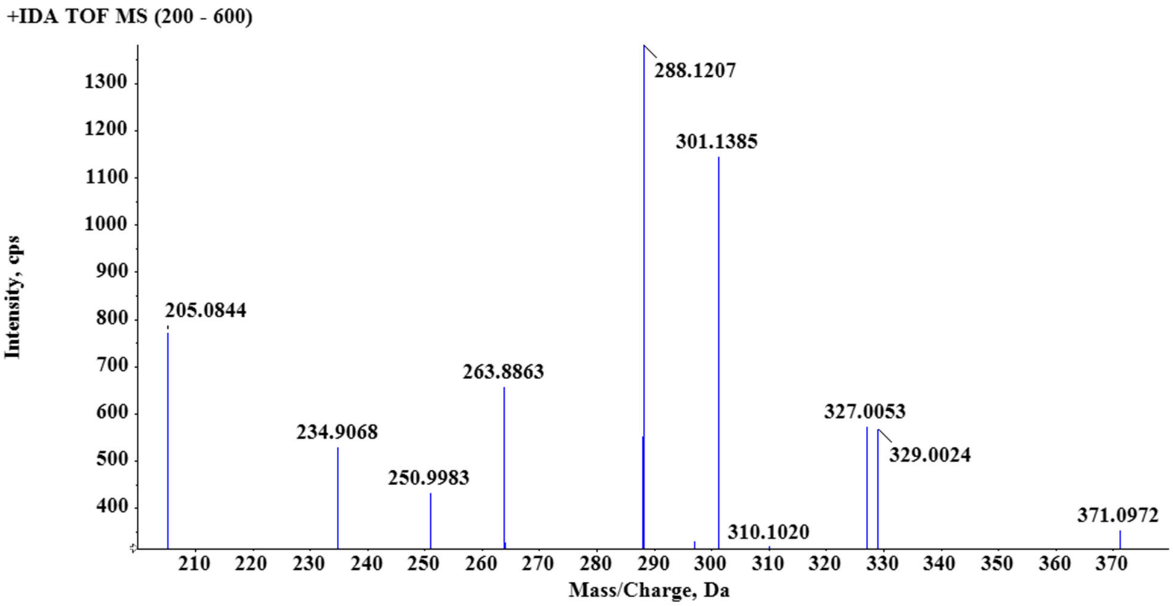

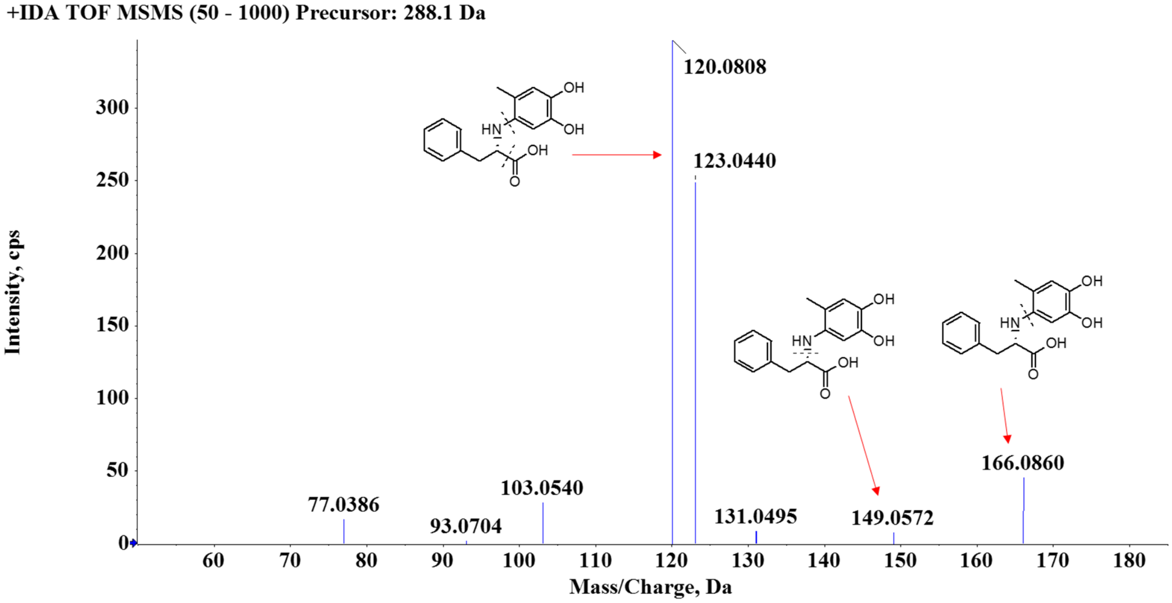

Figure S16. Mass spectra, proposed structures and corresponding fragmentation pathways of L-Phe-4-MC adduct.

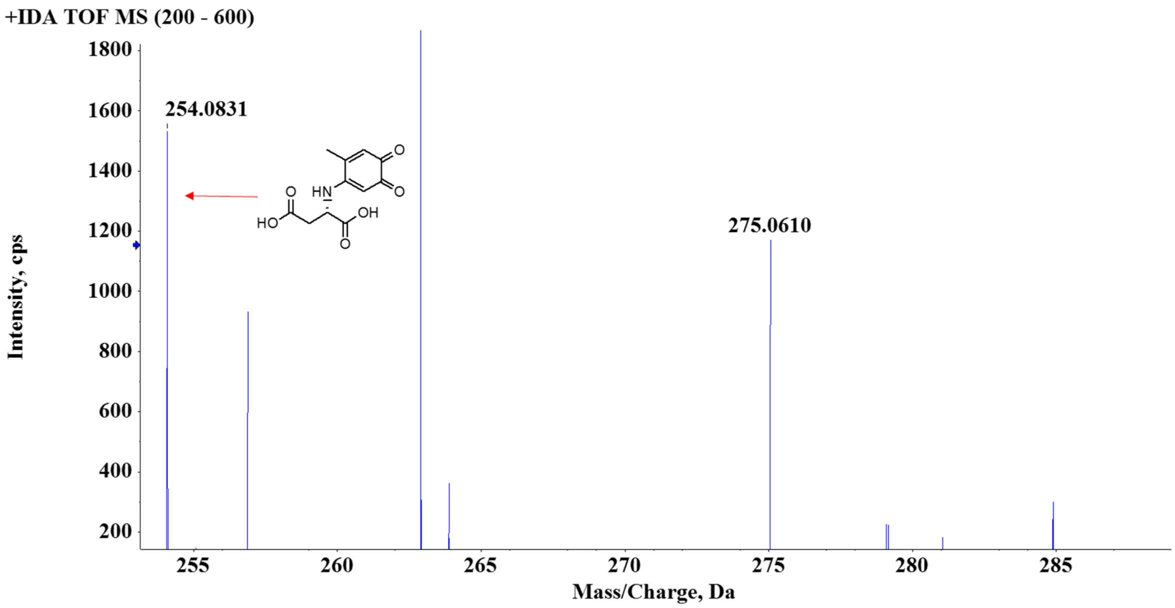
+IDA TOF MS (200 - 600)
254.0831
275.0610
Intensity, cps
Mass/Charge, Da
HN
HO
OH
O

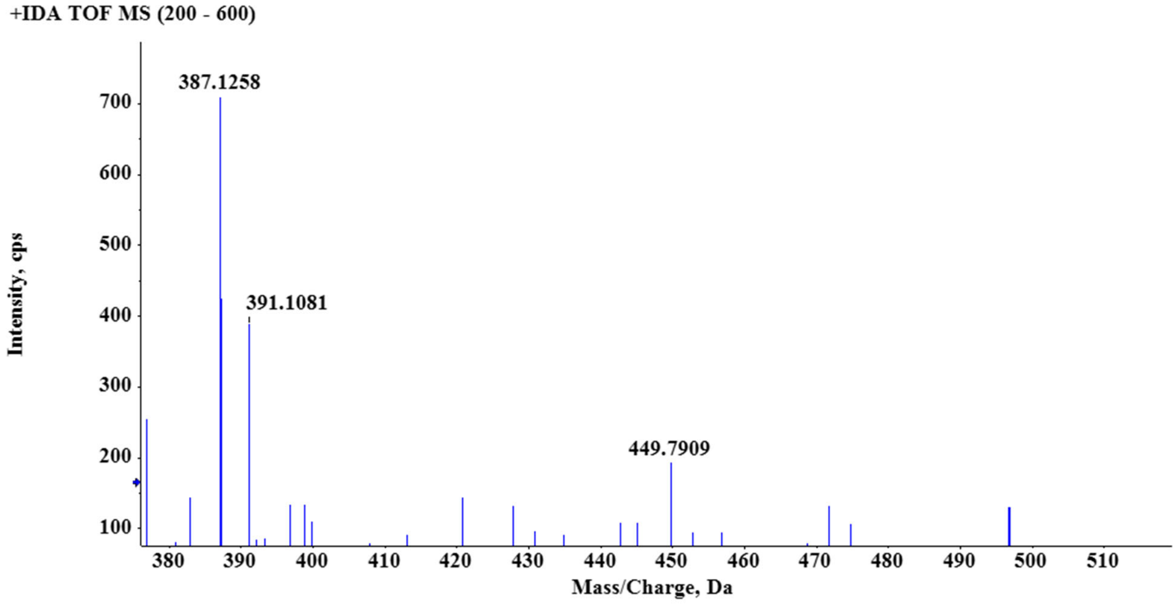
+IDA TOF MS (200 - 600)
387.1258
391.1081
449.7909
Intensity, cps
Mass/Charge, Da

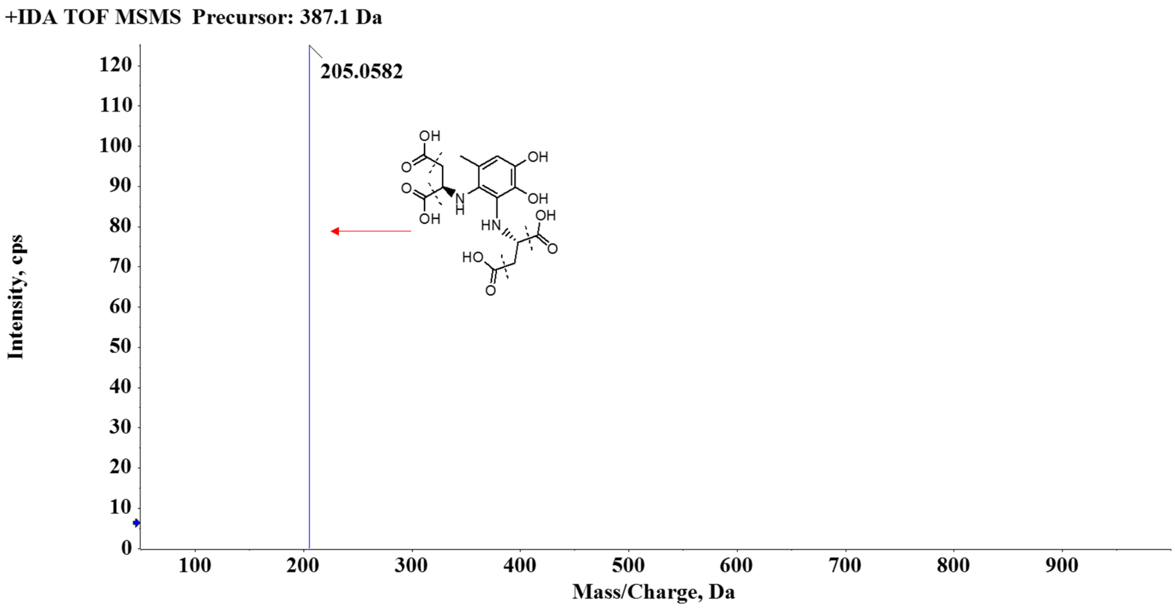

Figure S17. Mass spectra, proposed structures and corresponding fragmentation pathways of L-Asp-4-MBQ and L-Asp-4-MC-L-Asp adduct.

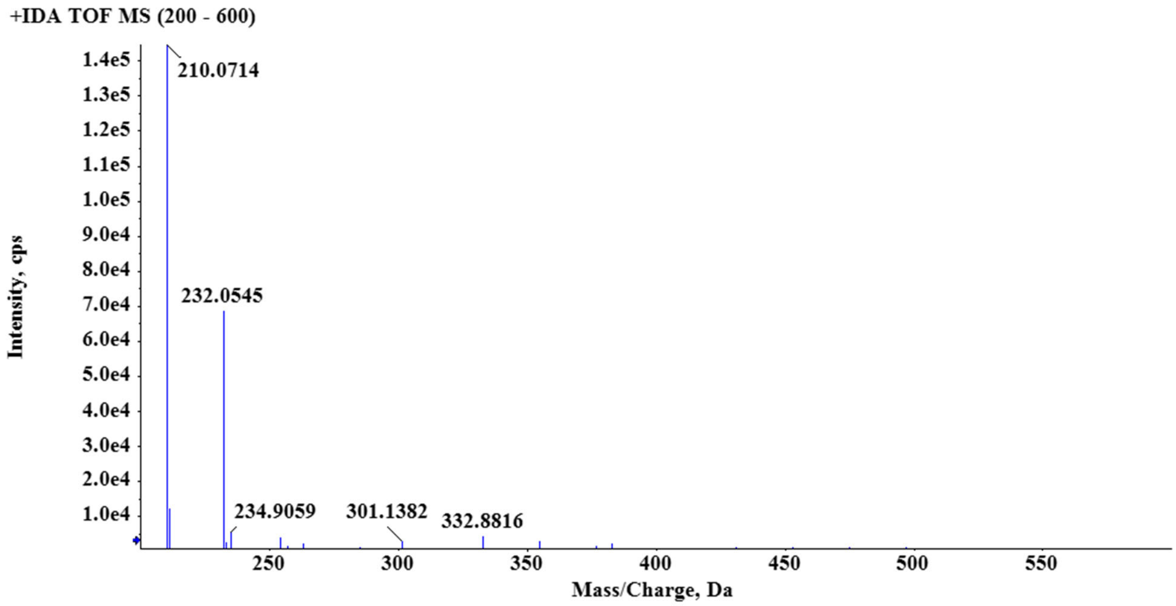

+IDA TOF MSMS Precursor: 210.1 Da

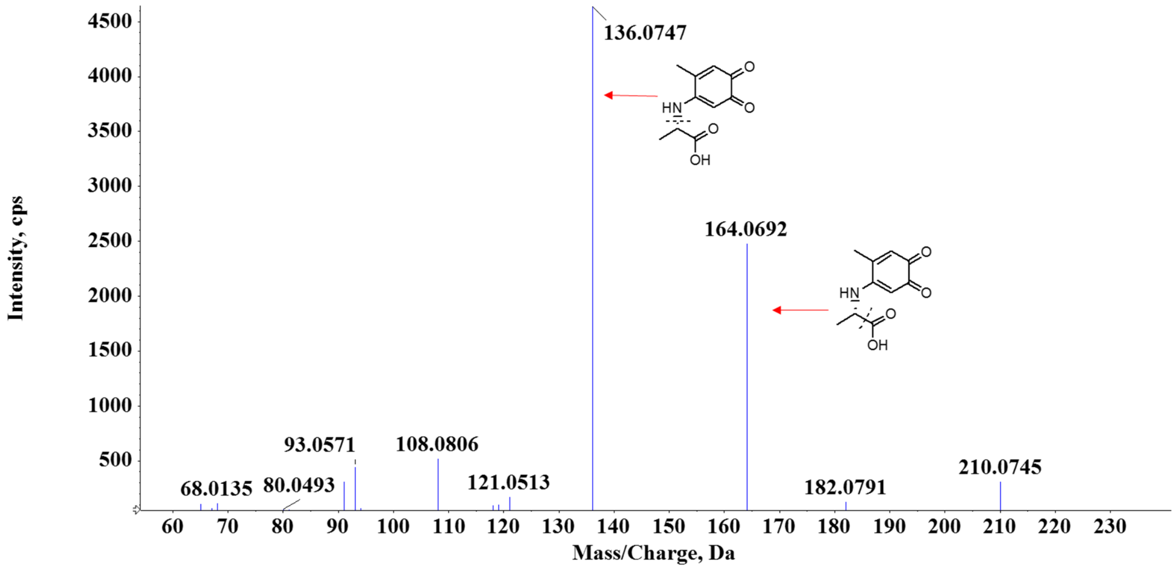

Figure S18. Mass spectra, proposed structures and corresponding fragmentation pathways of L-Ala-4-MBQ adduct.

+IDA TOF MS (200 - 600)

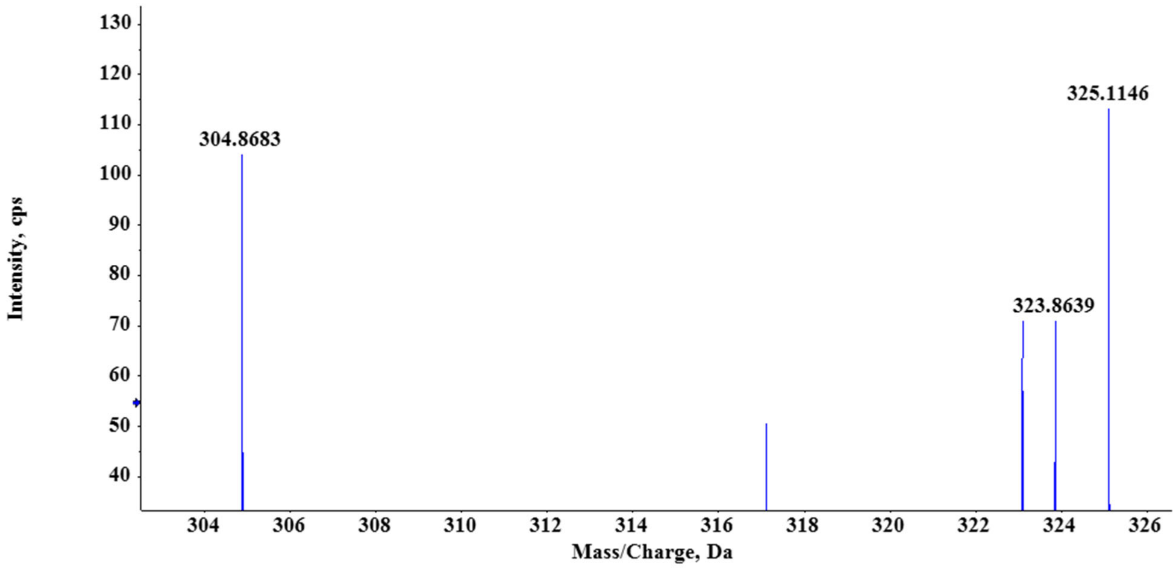

+IDA TOF MSMS Precursor: 325.1 Da

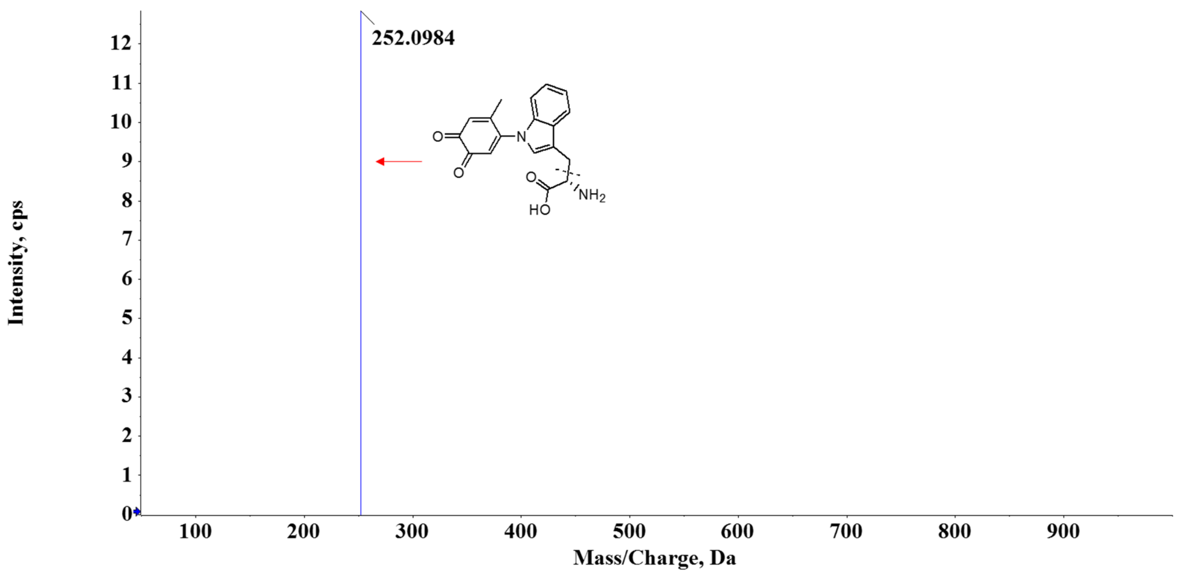

+IDA TOF MS (200 - 600)

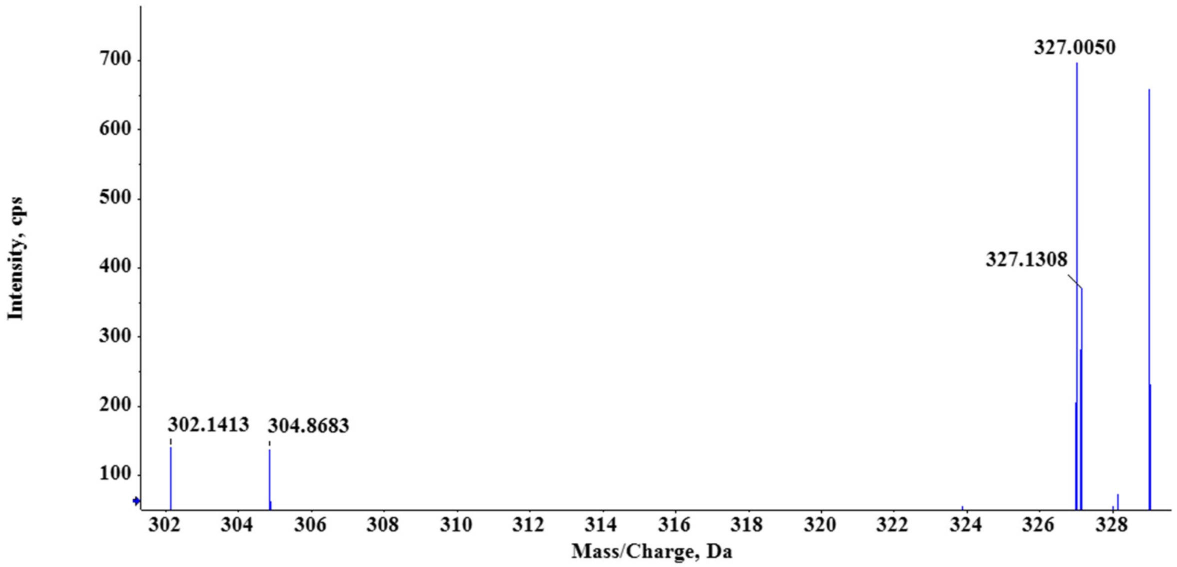

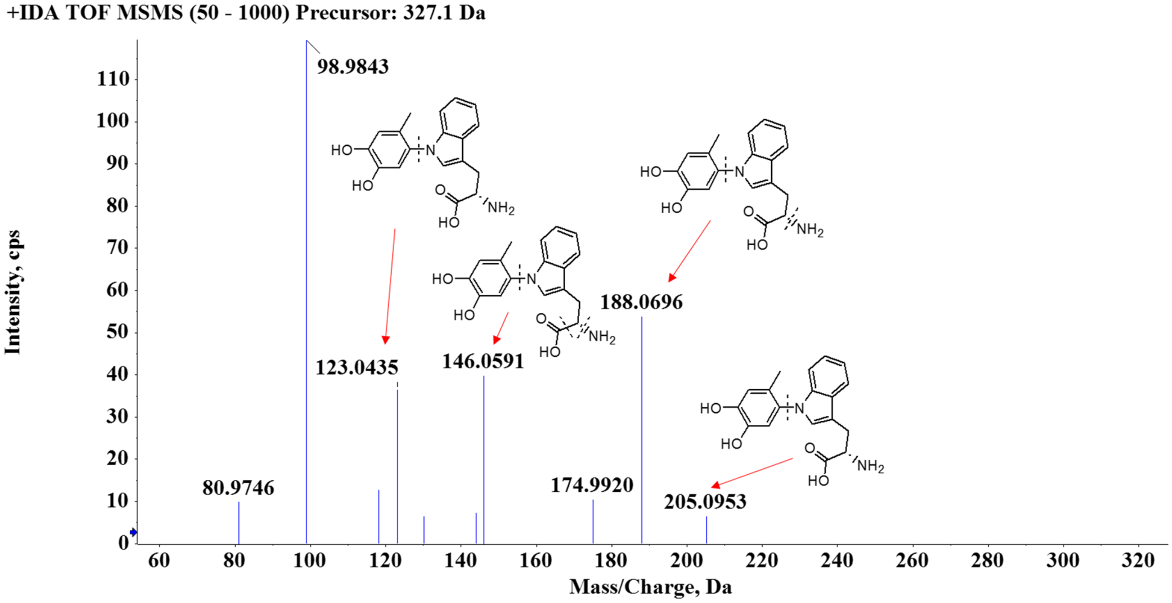

Figure S19. Mass spectra, proposed structures and corresponding fragmentation pathways of L-Trp-4-MBQ and L-Trp-4-MBC adduct.

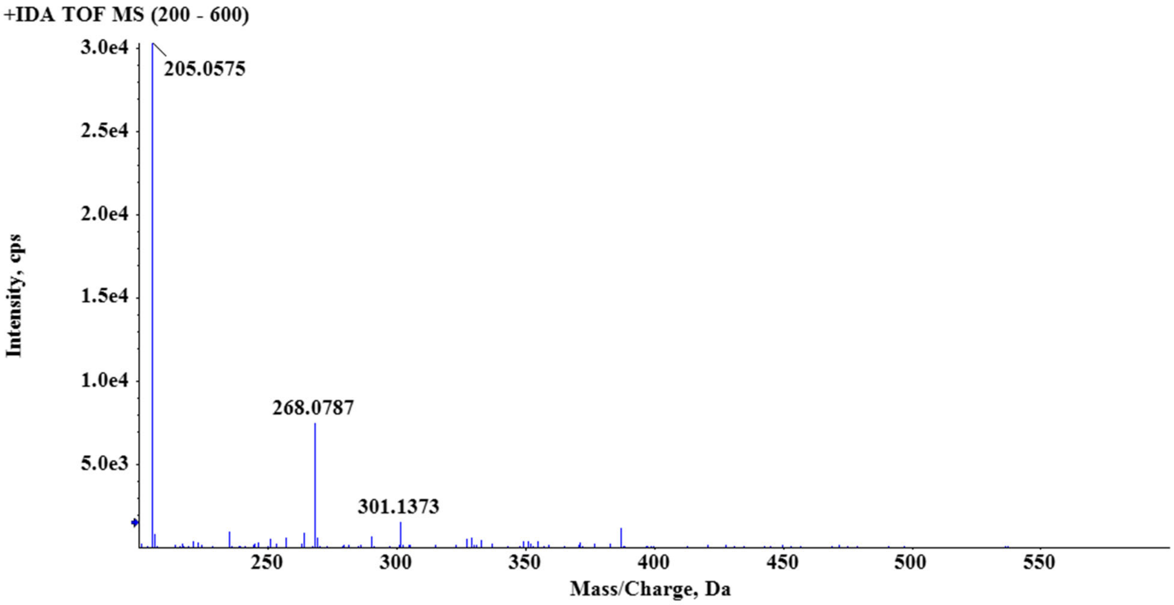

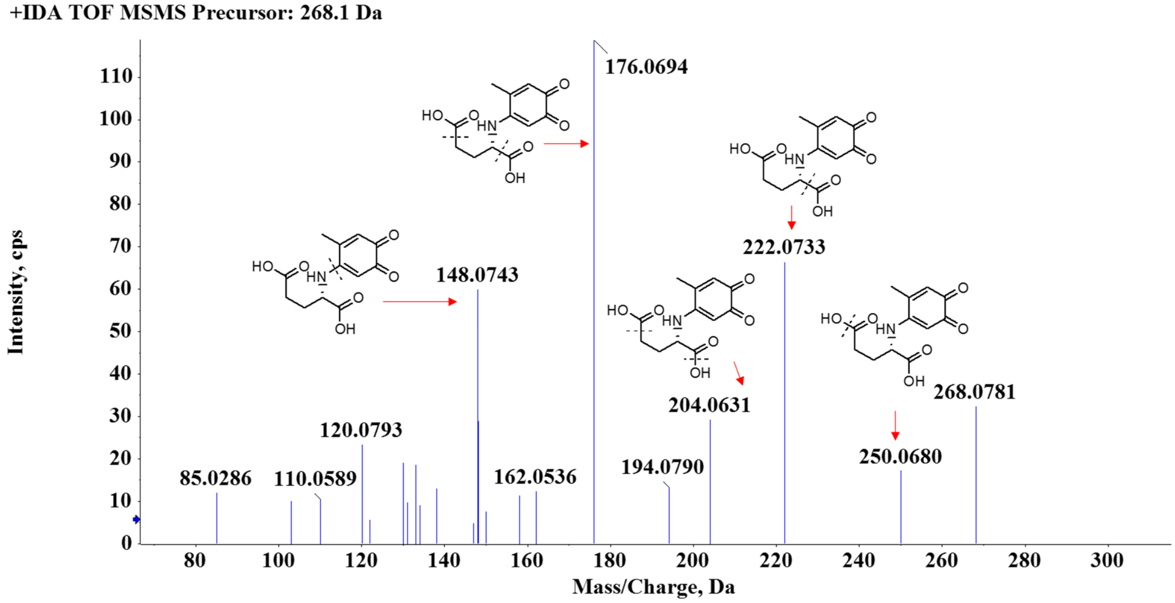

Figure S20. Mass spectra, proposed structures and corresponding fragmentation pathways of L-Glu-4-MBQ adduct.

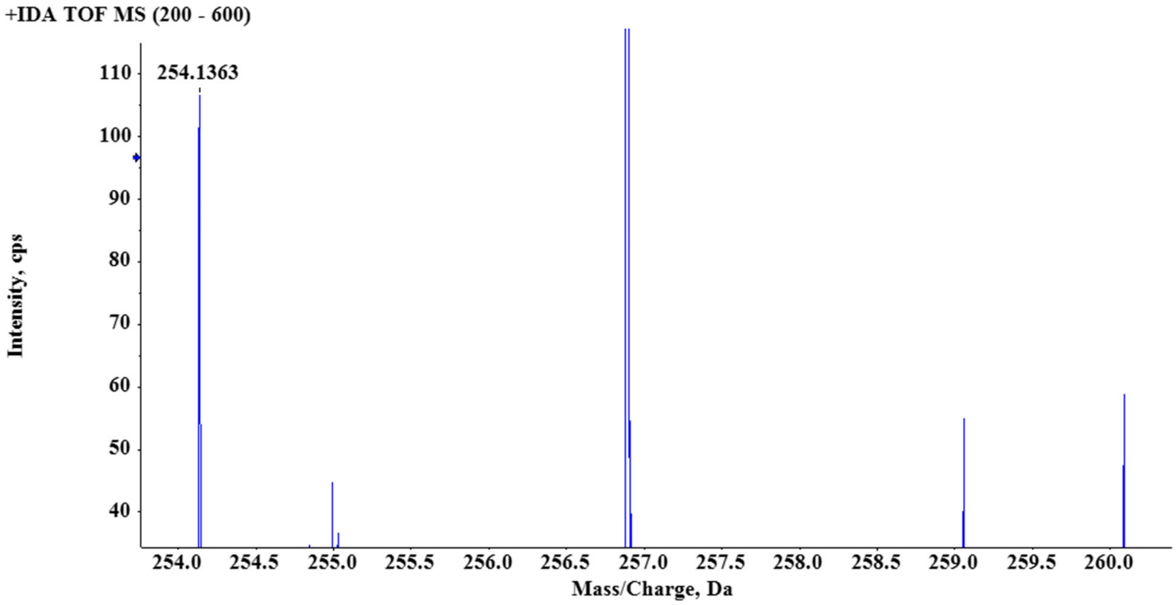

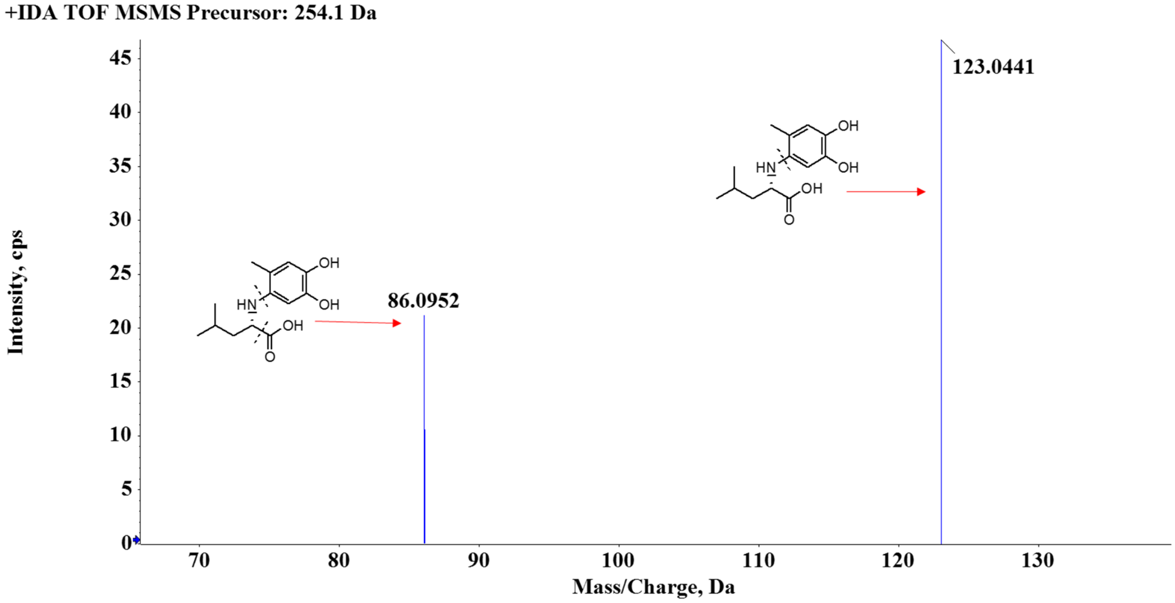

Figure S21. Mass spectra, proposed structures and corresponding fragmentation pathways of L-Leu-4MC adduct.

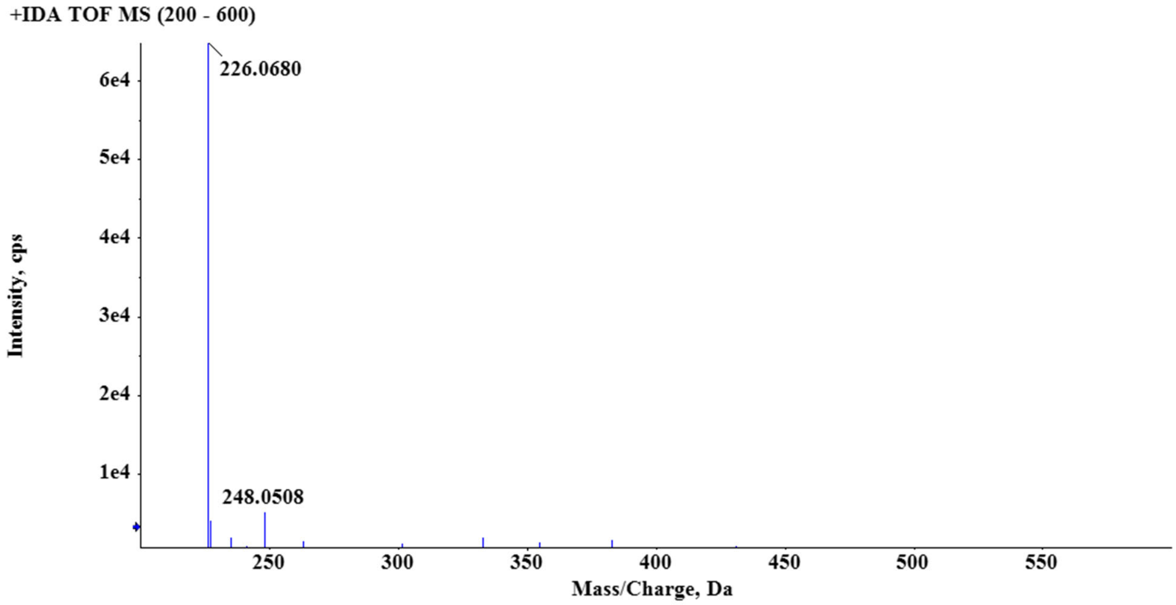

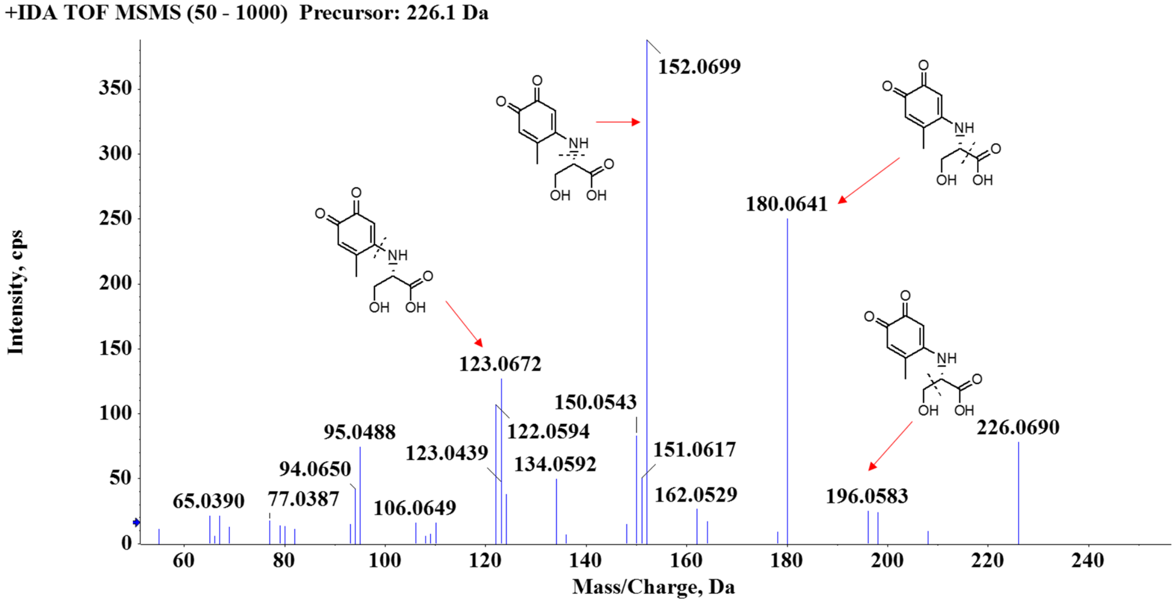

Figure S22. Mass spectra, proposed structures and corresponding fragmentation pathways of L-Ser-4-MBQ adduct.

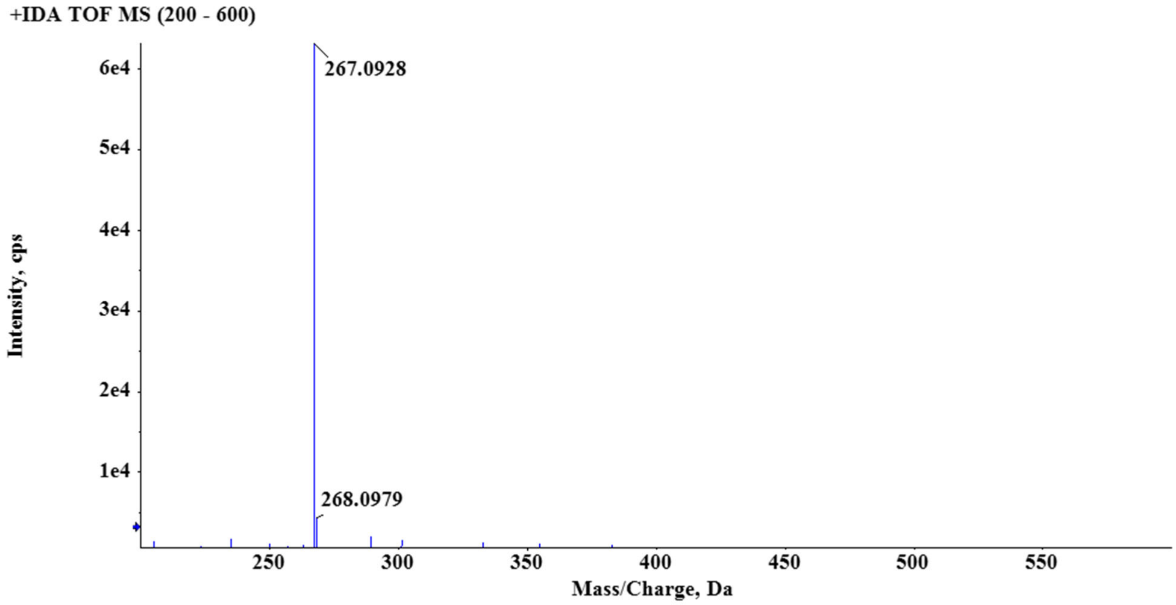

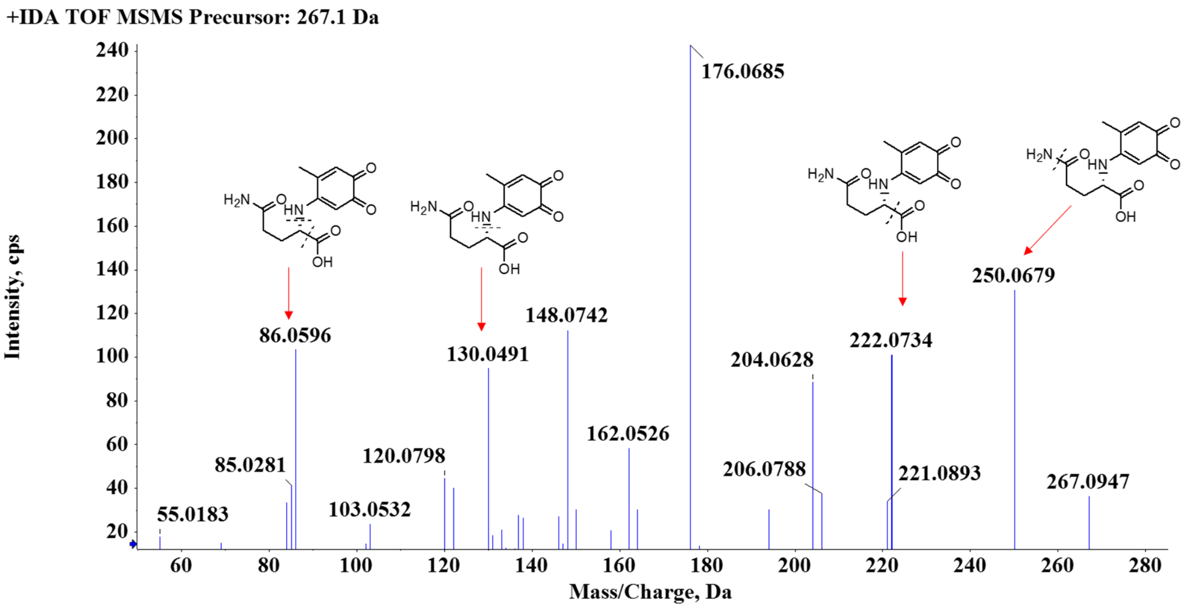

Figure S23. Mass spectra, proposed structures and corresponding fragmentation pathways of L-Gln-4-MBQ adduct.

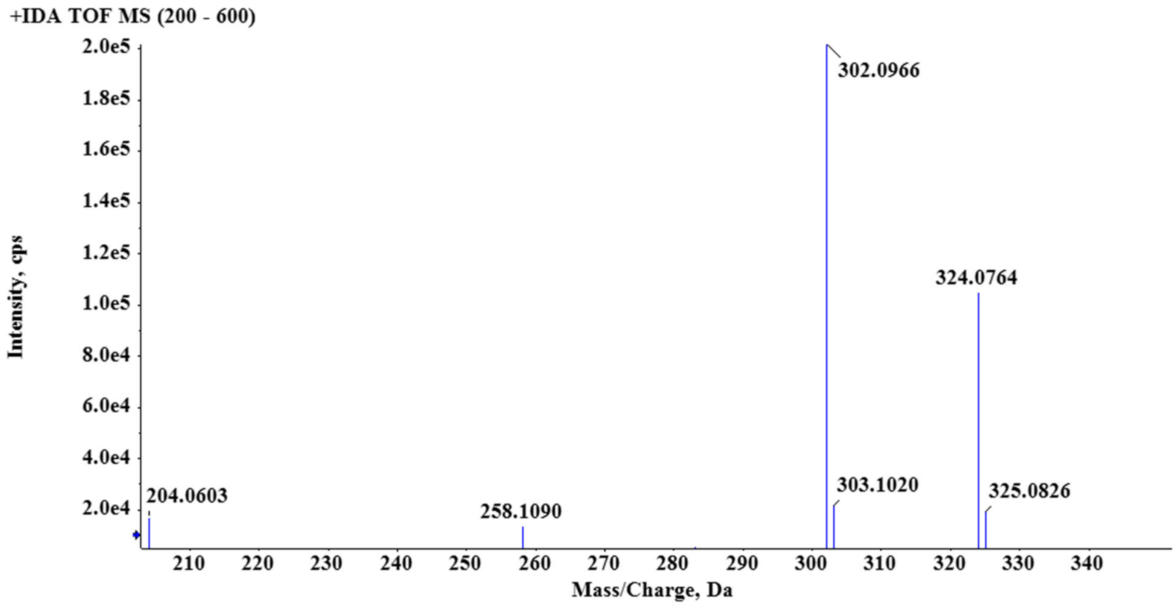

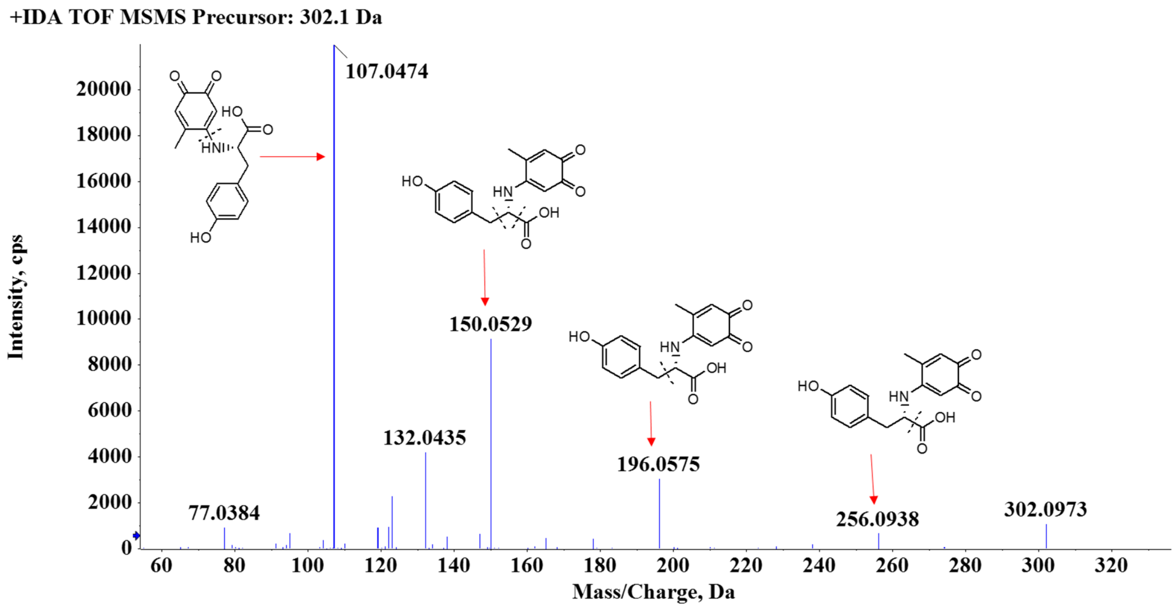

Figure S24. Mass spectra, proposed structures and corresponding fragmentation pathways of L-Tyr-4-MBQ adduct.
